# Exploring parental understanding of child sexual abuse and prevention as a measure for HIV prevention in Rwampara district

**DOI:** 10.1371/journal.pone.0269786

**Published:** 2022-06-30

**Authors:** Aloysious Kamukama, Rachel Luwaga, Rodrick Tugume, Margaret Kanyemibwa, Betrace Birungi, Obed Ndyamuhika, Diana Ampire, Timothy Nduhukire, Deborah Lowell Shindell

**Affiliations:** 1 Department of Nursing, Bishop Stuart University, Mbarara, Uganda; 2 Department of Pediatrics and Child Health, Kabale University, Kabale, Uganda; 3 School of Nursing, University of Nevada, Reno, Nevada, United States of America; Moi University School of Medicine, KENYA

## Abstract

**Background:**

Worldwide, more than 95 million children are sexually abused each year with children in sub-Saharan Africa experiencing sexual assault at higher rates than those in more developed areas. In Uganda, 20% of young people indicated that their sexual debut was non-consensual. The risk for transmission of HIV to children through Child Sexual Abuse is high because of greater mucosal tissue damage and the often repetitive nature of abuse. This contributes significantly to the burden of HIV in Uganda. Despite these risks, studies have shown gaps in active parental involvement in child sexual abuse prevention despite their being the primary protectors of children. Against this background we sought to explore parental understanding of childhood sexual abuse and prevention as a measure for HIV prevention in Rwampara District, South Western Uganda.

**Methods:**

A phenomenological study was carried out in four health centers that serve the communities of Rwampara district. A total of 25 (n = 25) parents or guardians of children aged 9–14 years were purposively selected to participate in the study. The participants were subjected to in-depth semi-structured interviews which were recorded, transcribed, and translated for thematic analysis.

**Results:**

Parents’ understanding of child sexual abuse was limited to penetrative sex between a man and a child. Three of the parents interviewed reported to have had children who had been sexually abused while one of the parents had been abused when she was young. The children reported to have been abused were female and were between 3-14years. We also identified gaps in the sensitization of parents regarding home-based prevention of child sexual abuse and psychological support for the victims of abuse.

**Conclusion:**

Our study shows that child sexual abuse exists in rural western Uganda. There remains a significant gap in the awareness of parents regarding the extent of sexual abuse, signs of sexual abuse, case handling, and psychological support for victims of sexual abuse. This significantly affects the capacity of parents as the primary protectors of children to identify and protect the children against the multiple forms of child sexual abuse.

## Background

Child sexual abuse (CSA) is an act where a child is forced or encouraged to take part in sexual activity. It includes penetration of the vagina, anus, or mouth by the penis, fingers or other objects. Non-penetrative sexual activities may include attempts to do any of the above listed as well as fondling with or without clothes on, exhibitionism, watching others engage in sexual acts and pornography [1].

Globally, over 95 million children experience sexual abuse annually [2]. The worldwide prevalence of CSA is estimated to range from 8 to 31% for girls and 3 to 17% for boys [3]. A recent review of the evidence suggested that rates of child sexual abuse were generally higher in sub-Saharan Africa than in many other parts of the world [2]. Other countries in sub Saharan Africa like Zambia registered 2396 cases of child defilement in 2012 [1]. In Uganda a great increase in defilement cases from 7,690 in 2011 to 14,567 in 2017 was reported to the Uganda Police force, however this may not be the actual number as many of the cases are not reported [4].

Child sexual abuse has short and long term sequelae which greatly predispose the victim to acquisition of human immunodeficiency virus (HIV). Short term sequelae happen because of the trauma from penetrative sex and the long term psychological trauma predisposes the children to risky sexual behavior including early onset of sexual activity and increased number of sexual partners as they mature [5, 6].

Worldwide, there are over 1.7 million children and adolescents living with HIV, the majority of them in sub-Saharan Africa [7]. Within sub-Saharan Africa, the disease is not evenly spread with West and Central Africa having the lowest incidence of adolescent HIV at 46 cases per 1,000 adolescents but in Eastern and Southern Africa, the rate is nearly four times that, 1.90 per 1,000 teens [8]. The HIV prevalence of young adolescents (10–14 years) in Uganda has been reported as 0.7% [6]. When evaluating the total number of HIV cases in children and adolescents, it is important to note that vertical transmission from mother to child has been declining rapidly over the last 10–15 years due to extensive outreach to pregnant mothers.

Currently, 9 out of 10 HIV positive pregnant women in Uganda are able to access antiretroviral treatment. This has led to a significant reduction in the rate of mother to child transmission of HIV. In 2015, the rate of vertical transmission of HIV was just 2.9%, exceeding the Global Plan milestone of reducing mother-to-child transmission of HIV among breastfeeding women to below 5% [9, 10]. Though the above have been achieved, child sexual abuse still poses a risk of increasing HIV infection in young adolescents as cases of defilement keep rising. While the incidence of HIV acquisition among sexually abused children is unknown due to weakness in reporting and data collection, there is evidence to show that CSA still poses a great threat in exposing children to HIV through physical trauma [11].

Several different CSA prevention programs have been implemented in different countries, through mass and social media platforms as well as through informal education by parents in home settings. These programs have been found to increase the child’s knowledge of CSA concepts and strengthen their intended responses [12]. Despite the implementation of these programs, there are several limitations to their effectiveness including; not knowing whether the children can transfer the information given by parents or other sources into protecting themselves from actual threat, appropriately disclosing when it occurs, and what parents ought to do to safeguard their children from sexual abuse [13]. As the primary providers of sexuality education in the formative years, parents play a significant role as a child’s protector. The provision of strong external barriers like a safe home environment and good communication enhance the ability of the children to recognize CSA, protect themselves from perpetrators and disclose when abuse has occurred [12, 13].

This study therefore provides both insight and understanding of parents regarding child sexual abuse and its prevention in rural -western Uganda. The findings of this study add to the existing literature on experiences and practices of parents in CSA prevention as an approach to HIV control and further research guidance.

## Methods

### Study design

A phenomenological qualitative study design was employed to explore parents’ awareness, understanding and experiences in regard to child sexual abuse and prevention as told in their own words.

### Study setting

The study was carried out in four government-owned health centers in Rwampara district, South-Western Uganda. The setting was selected because 35% of all sexual abuse cases reported at the Rwampara district police station in 2020 were for child sexual abuse (personal communication from probation office). This provided a high concentration of families who may have experienced child sexual abuse.

### Study population

The population for this study included all parents of children and adolescents living in Rwampara, South Western Uganda. However because it was not feasible to study the entire population, a purposive sample of 25 participants was recruited from the selected health centers in Rwampara district. The participants were chosen because they were guardians or parents of children aged 9–14 years. Children of this age group were selected because it is at this age that children tend to seek more emotional attention, predisposing them to sexual exploitation.

### Sample size estimation

The study sample size was a maximum of 25 participants as per the recommendations for phenomenological studies [14].

### Sampling method

Designated nurses at each of the health centers purposively identified potential participants who met the inclusion criteria (having children 9–14 years old). For the health centres that service a large number of patients i.e. Kinoni HC IV and Bugamba HC III a total of 15 participants were selected (8 from the outpatient department and 7 from the ART clinic). For Mwizi HC III and Ndeija HC III a total of 10 participants were selected (6 from each of the outpatient departments and 4 from the ART clinics).

### Data collection

#### Data collection tools

An in-depth interview guide with open ended questions was developed to guide the discussions. The questions included prompts asking if the participants had ever heard about CSA, if they could define CSA, if any of their children had ever experienced sexual abuse or if they believed it existed in their communities. Other prompts included what the family was doing to prevent child sexual abuse in their homes and in their communities. The prompts were chosen to help determine the parents understanding and awareness about child sexual abuse and if they understood how to prevent it.

#### Data collection procedures

Once consent was obtained, all participants were interviewed one on one by trained research assistants who were fluent in the local language. The interviews were carried out in the local language to obtain the best quality data. The interviews occurred in a private area of the health center and lasted for a maximum of 30 minutes. Each interview was audio recorded and note books were used to capture the specific aspect of the interviews.

After the interviews, the recordings were transferred onto a computer and backed up on a flash drive. All recordings were then transcribed by the research assistants that interviewed the participants to ensure accuracy of both content and intent. The transcribed documents were then given to experienced translators fluent in both the local language and English. Once the documents were translated to English, the researchers who were fluent in both the local language and English listened to the interviews and compared them to the translated English version to ensure accuracy. Once the researchers were satisfied with the accuracy of the translated English versions, the documents were sent for analysis.

### Data analysis

To develop the key themes present in the interviews, the translated English versions of the interviews underwent thematic analysis. Thematic analysis was selected for the study as it allows for analysis and understanding of the meaning of multiple coded responses from interviews in their true context [15].

The translated scripts were read by each of the three researchers independently to identify key responses related to the objectives as well as unique responses to the prompts.

After locating repetitive phrases, responses that included the phrases were highlighted and color coded in each interview. The color coded phrases were then grouped into categories and the categories organized into themes. During this analysis, themes directly related to the study objectives were identified. Additionally, emergent and new themes were also developed. The themes were then reviewed by the team for accuracy and to verify that they represented the true account of the participants.

### Reliability and validity

To eliminate individual researcher bias, each of the three team members (AK,RT and RL) were responsible for reading the translated scripts, as well as independently coding and generating meaning out of the data. The individual data was then compiled by the three researchers and evaluated for consistency. Any analyses that were conflicting or ambiguous were discussed amongst the researchers and interpretations clarified until there was congruence among all three researchers.

### Ethical considerations

The Mbarara University of Science and Technology Research Ethics Committee reviewed and approved the proposal (MUREC-27/11-20). Regulatory approval was obtained from Uganda National Council of Science and Technology. Administrative clearance was obtained from the District Health Officer and written informed consent obtained from each participant.

To ensure psychological support of the parents whose children experienced sexual abuse, the researchers arranged for the local clinic staff to identify counselors available to provide psychological support after the interviews should it be needed. Through the interview process however, we discovered that regarding the cases where the children had been sexually abused, reports had already been made to police, the children and their parents had also been counselled by the local health centre counsellors since the incidences had occurred more than a year prior to the interviews.

## Results

### Demographic characteristics of the participants

Majority of the participants (76%) were females, age range was 31–59 years, and they were predominantly farmers. Three of the parents reported to have had children who had been sexually abused while only one of the parents had been abused when she was young. The children reported to have been abused were female and were between 3-14years. For more see Table 1 below.

**Table 1:** Demographic characteristics of the participants (n = 25).

Variable	Categories	Frequency (%)
**Age (years)**	18–28	1 (4)
	29–39	12(48)
	40–50	5 (20)
	>50	7(28)
**Gender**	Male	6(24)
	Female	19(76)
**Occupation**	Farmer	18(72)
	Small Scale business	4(16)
	Others	3(12)
**Formal Education level**	Primary Level	17(68)
	Secondary level	4(16)
	No formal education	4(16)
**Marital status**	Married	16(64)
	Single	6(24)
	Widow	2(8)
	Widower	4(4)
**Relationship with the child or children**	Father	6(24)
	Mother	12(48)
	Caretaker	7(28)

### Themes and subthemes

Responses from the participants were divided into three major areas: understanding and awareness of parents regarding child sexual abuse, experiences of parents regarding child sexual abuse and experiences and practices of parents in child sexual abuse prevention. Within each area, multiple sub-themes were generated to better explain the phenomenon.

### Area one: Understanding and awareness of parents about child sexual abuse

Themes for this area emerged from the responses of the parents and caretakers when prompted to explain their understanding of child sexual abuse and to what degree they were aware of its occurrence.

Overall, participants expressed differing views regarding what the term “child sexual abuse” meant. Some respondents cited that this phenomenon only included a child sleeping with a man or the act of using a child to perform sexual acts. There were others who noted that child sexual abuse included children having sex or performing sexual acts with each other. Parents felt that this act was learnt from peers as exemplified in the quotations below.

“*It is using children in sexual acts*.” ***IDI 10, female 9/4/2021***

“*It is sexual intercourse*” ***IDI 03, female 7/4/2021***

“*One day I was at the hospital and some people brought in very young children that they were having sex, they were three boys who were having sex with one girl*, *They were like eight, nine and ten years yet the girl was only three years old*, ***IDI 03, female 7/4/2021***

“… *at times children copy these acts from their peers/friends, When they hear what their friends have done, they will also want to do it*” ***IDI 03, female 7/4/2021***

There were several predisposing factors to child sexual abuse mentioned by the respondents including; children having access to pornographic material on their parents’ phones at an early age. Additionally, a lack of privacy at home was also mentioned where the children are subjected to watching their parents/caretakers having sex and may want to do the same. Parents also mentioned that mistreatment at home including denying children livelihood and food can cause many of them to be lured into sexual activity to meet their basic needs as mentioned below.

“*They can see grown-ups having sex. In this modern generation you find people having sex in presence of children especially in bars*. *This exposure makes the children eager to do what these people are doing*”, ***IDI 03 female 7/4/2021***

“*Yes, very young boys copy their fathers*… *you hear them saying they are trying to do what their fathers do, I think somehow how they watch them*” ***IDI 03 female 7/4/2021***

“*Now these phones we have, the child can steal it from you and watch pornography*.” ***IDI10 female, 9/4/2021***

“*Our step mother was mistreating us, our father was rich but she could deny us food and lock us inside the gate. Our parents had a hotel and lodges but you would find us eating bones and those men who could come in to use the toilet had a chance to use us sexually*. *I would be rushing to have sex with a man so that he could leave me with some money*.” ***IDI 2 female, 6/4/2021***

The parents and caretakers identified their sources of information regarding child sexual abuse to be fellow parents who had either experienced sexual abuse themselves, from their children, their neighbors, radio programs and from unauthenticated sources or “rumors” in the community.

“Some *parents told us that their house boy (domestic worker) after working went into their house and raped their daughter*. *When she screamed neighbors came to her rescue and found the boy dressing up*. *He was then arrested*. ***IDI 23*, *female 06/04/2021***

“*When we saw the children coming while they were not walking properly, we rushed them to the health workers*. *Then we went to report and the girls mentioned the name of the person who had raped them, we found out that it was one of the men that stayed in this village so they arrested him and imprisoned him **IDI 18,male 6/04/2021***

“*I came to know about them from the radio and also being sensitized*. *You can hear that a father has raped his daughter or you hear that a girl was raped*.” ***IDI 22, male 6/04/2021***

### Area two: Experiences of parents regarding child sexual abuse

Some of the parents reported having been abused themselves in their childhood. One participant shared that she was forced into early marriage after being sexually abused because the parents lacked options to solve her abuse case.

“*Even me myself I wasn’t supposed to get married soon because we were not poor but because of our step mother mistreating us*. *She would lock us up without food. When a man would come into the lodge and calls me, I would rush to have sex with him for money. I ended up in this mess because of hunger*”**.” *IDI 02, female 7/04/2021***

Parents also explained that their own children had been abused. In some instances the perpetrators were familiar to the parents—that is fathers, uncles, employees or cousins of the children. In other instances, they were not known. Some of the experiences were gained from stories that were being shared in the community and in the neighborhood of the parents

“*Personally*, *I experienced it with my own child*. *I had sent my child to go and fetch water for me from the well which was somehow very far from our home*, *so when she delayed to come back*, *I sent her brother and I told him to go and look for her*. *When he reached there*, *he found his sister yelling and crying all over*, *after being raped*. *Her brother came back carrying her and that is when I saw it with my eyes but I used to just hear about it from others”* ***IDI 15 female*, *9/04/2021***

“*Yes, it has happened. Even my elder daughter experienced it when she was three years old*. *A casual laborer who was working at my in laws’ home got used to my children. He had stayed there for three to four years but that act couldn’t be imagined, you couldn’t even think that it could happen but it did*.” ***IDI 04 female 7/4/2021***

“*I was unaware that my daughters had been raped*. *One was eight years while another was six years old. Eh!! They were raped on the same day. When we saw the children walking with difficulty, we rushed them to the health workers. The girls reported that the man who raped them stayed in the same village. We reported the case to police, he was arrested and imprisoned*” ***IDI 18, male 6/04/2021***

“*A motorcycle rider was caught by his wife after defiling their daughter*. *She found the girl bleeding. The girl was taken to the hospital, given medication and she recovered. Fortunately her father was not HIV positive but he injured her*.” ***IDI 09 female 9/4/2021***

The process of handling the abuse cases was also an important area highlighted by the parents. If the victims were taken to hospital, they be tested for HIV and given post exposure prophylaxis.

“*I brought my children here at the hospital for a medical examination because the man who raped them was HIV positive*. *They tested them twice and found that they were not infected and were safe*” ***IDI 18, male 6/04/2021***

“*Yes, she was three years and* …….*it was Saturday when it happened but I knew about it on Sunday evening and I brought her to the health centre on Monday. That’s when they told me that she has been raped but a good chance she was not injured severely. I was given some tablets and we went back home because they told us that the child was safe. Reaching home, her father took her to Mbarara hospital. She was admitted and given all necessary medication for two weeks. She was also tested for HIV and turned negative but we were still given medicine of three types; tablets and syrup that was to be administered in same hour. This lasted for one month and I was advised to take her for HIV testing when she makes five years. When she made five years, I took her back and she was still fine*.” ***IDI 4, female 7/04/2021***

Parents also cited frustration regarding handling of cases at police. Parents mentioned that punishments varied for the different perpetrators; in the instances where the children were having sex with their peers, the children were tested for HIV and subjected to severe caning since they were minors. Some of the adult perpetrators were subjected to severe caning and released if they were HIV negative, while the majority stayed in prison. Other cases were resolved silently by the family so as to protect the name and integrity of the family as mentioned in the quotations below.

“*I had my daughter she had finished Primary seven and eloped with a boy*. *I went to the subcounty Rugando where I made a report that my 14 year old was lost. The officer in charge just said that ‘the child is old and she going to embarrass you. If she still loves her boy, leave her to get married because she has already spent one week away from your home, you can’t bring her back home and she is a woman now she does not want to go to school*’.” ***IDI 20, female 7/04/2021***

“*The children were forgiven, given strokes (beaten with sticks/canned) and since they were not HIV positive they let them go*., ***IDI 03. Female 7/04/2021***

*After the perpetrator was caught, we went to the chairman and we had him arrested*… *finally he was put in prison, **IDI 15, female 9/04/2021***

Parents further cited that some of the consequences of child sexual abuse they witnessed were ranging from early pregnancies, where the parents of the girls who were abused had to take responsibility of the girl or the young man who made her pregnant had to care for the girl yet he was also a child, to HIV acquisition and death.

“*The one I know is not from my village but is from this sub county of Bugamba, an old man sexually abused a girl several times*. *One of the Village Health Team members (VHT) found her and brought her to this health centre. She was tested for HIV and was found HIV positive. We got to know that the man who had abused her had recently died. The child started ARV’S (Anti Retro Viral drugs).**” IDI 03, female 7/04/2021***

“*An HIV positive forty year old man defiled a minor of thirteen years, when the child was eventually tested for HIV, she was found positive*.**” *IDI 07, female 7/04/2021***

“… *there was a parent who hid the daughter at home after she had been raped and we later saw the girl pregnant*. *That’s how we found out that she had been raped.**” IDI 25, male 6/04/2021***

### Area three: Experiences and practices of parents regarding CSA prevention

Parents cited practicing child counseling, guiding children to avoid certain peer groups, monitoring, restricting of children’s movements and sending them to boarding school as some of the means of preventing their children from being sexually abused. Parents also cited the need for more community sensitization regarding child sexual abuse and its prevention as mentioned in the quotations below.

“*I tell them that we are in difficult times so they should avoid people who try to lure them by giving them money*.” ***IDI 23, female 9/04/2021***

“*I discourage her from bad companies and tell her that in case you join them you will die soon before you are eighteen years or get pregnant*” ***IDI 06, female 8/04/2021***

“*You teach the child, you tell him, that a person should not call you and subject you to bad acts, or if it is a girl you tell her*, ‘*if you meet a boy and see things that are not good, tell me don’t fear me’, you say ‘I have seen this or someone has told me this and that’,” **IDI 19 female 9/04/2021***

“*Talk to the child, because when he/she has known about sexual abuse when it is still early, he/she will grow up scared about it*. *Take care of her and provide for her needs so that she does not envy her peers and get lured into sexual acts “, **IDI 02, female 04/2021***

“*We protect them by staying with them at home and if it means going to dig with them, then go with them. Just make sure that the children are busy all the time but if you spend all your time in clubs drinking alcohol then you are not protecting them*.” ***IDI 21, female 6/04/2021***

“*We have a very big problem, but these boarding schools came to help us because when I child is in school she’s safe from these evil doers especially like us who are always in town and the children are home alone and the child has reached adolescence stage, they are only looking forward to destroying that child*” ***IDI 2, female 6/04/2021***

“*There are usually monthly seminars where the youth and parents are sensitized about sexual violence*. *Even the VHT usually advice parents and the youth on what to do” **IDI 23, female 9/04/2021***

*“I would call upon the leaders to continue sensitizing us on how to protect the lives of our children because personally I could be protecting myself but there are some people who don’t know what to do*.*”* ***IDI 21*, *female 6/04/2021***

## Discussion

### Parental understanding and awareness of child sexual abuse

As noted previously, child sexual abuse is an act where a child is forced or encouraged to take part in sexual activity. It includes penetration of the vagina, anus, mouth, by the penis, fingers or other objects. Non-penetrative sexual activities may include attempts to do any of the above listed but also fondling with or without clothes on, exhibitionism, watching others engage in sexual acts and pornography [1]. Many of the parents limited their understanding of child sexual abuse to sexual intercourse with a minor involving penetration. None of the parents interviewed mentioned fondling or subjection of children to pornographic material as abuse.

This makes it very difficult for a parent to understand the extent to which their children can be or are being abused. This lack of understanding can be attributed to the fact that in the African culture, sex is not a topic that is easily shared. Although many parents find it necessary to have this talk with their children, many are not comfortable discussing such issues with anyone, especially their children [16].

The increased level of poverty in rural areas contributes greatly to the exposure of children in these settings to sexual abuse. Living conditions such as homes where children and parents or other adults sleep in the same room without privacy expose children to the viewing of sexual acts. This creates an early awareness about sex and may ignite curiosity on the part of the children to carry out such acts. As some of the parents mentioned, the boys want to emulate what their fathers are doing.

Lack of food and unmet basic needs were cited as major areas that cause children to be lured into sexual abuse. When children have no food, they may be forced to give sex in exchange for money, as was commonly mentioned by the respondents in this study. Mistreatment from step-parents where children were denied food and care causing them to fend for themselves creates a room for sexual exploitation [5, 6, 17].

Historically, in African cultures, it was known that children were the property of the community and therefore there was community involvement in the welfare of the children. Development as well as increased levels of mistrust in communities have changed this situation leading to multiple sexual abuse cases going unreported or under-reported because people are accustomed to “minding their own business”. Parents noted that sometimes children are seen touching each other in sexual ways but since this may create enmity between families if reported, those that usually observe these actions choose to keep quiet over the matter. This is probably one of the reasons why the cases for child sexual abuse are under reported [18].

### Experiences of parents regarding child sexual abuse

Child sexual abuse has short and long term sequelae which greatly predispose the victim to acquisition of HIV [5, 6, 17]. Parents reported being aware of situations where the children who were abused were actually infected with HIV by the perpetrator of the abuse. Another unfortunate consequence of child sexual abuse that was mentioned by the parents was early marriages. Many of the young girls who are forced into early marriage also do not get psychological therapy after the abuse, perpetuating the cycle of control and abuse. This observation has also been made in several other studies [5, 6, 17].

One of the difficulties cited by parents in this study was that when abuse was reported, the handling of the cases did not always lead to justice. Some of the perpetrators went un-punished, others were briefly jailed and then released. If it was children abusing each other, there were few repercussions. Reports in Uganda confirm that many of the perpetrators go unpunished leaving the children distressed because of lack of justice [4]. There is lack of psychological support for the children who are abused and the parents don’t know how to handle such situations. This hinders a child from coping positively from the abuse and predisposes them to further sexual abuse. Creating psychological stability for the children who have been abused is very important to enable them make right choices in future and not blame themselves for what happened to them [4].

### Experiences and practices of parents on CSA prevention

Other studies regarding parent-involved prevention of child sexual abuse established that a parents’ perceptions of CSA and their communication with children about CSA prevention can positively affect children’s prevention knowledge and self- protection skills [19].

Our study found that parents attempt to counsel their children about ways to protect themselves from being abused such as telling them to stay away from men, to run if they see a stranger and to be content with what they have been provided by their parents so that they are not lured into exploitive situations. The gaps in parent-child communication realized in our study were that the parents are not specifically telling their children what sexual abuse is, have limited communication with their children and are not being watchful as many stated that they must leave children on their own when they go to work.

There is a need to create age appropriate materials for parents to help their children understand what sexual abuse is. Some of the parents appealed for more parent education on the subject as they lacked any formal training to be able to help their children. The importance of giving parents formal training in a bid to boost the fight against CSA has been reported in past studies. It has been shown that parents who receive training in CSA prevention and/or recognition of signs of abuse were significantly more likely to report suspected CSA compared to those without such training [20]. Studies also found that parents-involvement in education especially regarding sexual abuse had a positive effect on children’s CSA prevention knowledge and encouraged disclosure of abuse when it happened [21].

## Conclusions

Our study shows that child sexual abuse exists in rural western Uganda. There remains a significant gap in awareness of parents regarding the extent of sexual abuse, signs of sexual abuse, case handling and psychological support for victims of sexual abuse. This significantly affects the capacity of parents as the primary protectors of children to identify and protect the children against the multiple forms of child sexual abuse.

### Limitation

The main limitation to this study was the potential for interviewer bias. This limitation was mitigated by directly following the probing questions in the interview guide and seeking for clarity from participants if the responses were not clearly understood. The analysis was also done by three members of the team independently and then the interpretations were compiled to come up with the reported themes.

Though the incidences happened at least 1–2 years prior to the study the participants were able to give a full account of the abuse because it happened to their own children. The gravity of the incident left an indelible mark in the lives of the parents. The memory was so fresh in their minds as if it had occurred in the recent past. This therefore mitigated the limitation of recall bias.

### Recommendation

Parents desire education so that they can protect their children. Community based trainings for both parents and children should be held to create awareness about the scope of sexual abuse as well as mitigation measures. The creation of parent friendly material for home based sexual education and child sexual abuse prevention is necessary to increase the ability of parents to teach their children at home.

Creation of centres for CSA survivors will go a long way in providing psychological support to victims of CSA in the community.

Refresher training for members of security services involved in child protection on the scope of CSA and its devastating effects on the victims will help in improving the way cases of child sexual abuse are handled.

This study recommends further research on the magnitude of child sexual abuse in Uganda, its effects and interventions if any that have been made to curb it.

## Supporting information

S1 File (ZIP)
