# Supplementary material for: Exploring parental understanding of child sexual abuse and prevention as a measure for HIV prevention in Rwampara district
Source: PLoS One. 2022 Jun 30;17(6):e0269786. doi: 10.1371/journal.pone.0269786 (PMC9246133; doi:10.1371/journal.pone.0269786)
Supplement: S1 File — (ZIP) [file pone.0269786.s001.zip › Child Sexual Abuse data final.docx]

**Translation 1**

**I:** You are welcome and like I had explained to you, our discussion today will be about challenges faced by parents to prevent children aged 9 to 11 years from involving themselves in sexual acts as a preventive measure against HIV. For you have you heard involving children in sexual acts?

**R:** Like on radio, in the village you hear that a man has raped his own child and does not know them, you hear them from the radio, you feel so bad. but I have not seen it in our village or that I hear about him. but you hear from other villages when he is there hearing them being talked on radio.

**I:** So when someone tells you using a child in sexual acts how do you understand it?

**R:** Involving in sexual acts I think its promiscuity.

**I:** Sexual acts involves what and what?

**R:** Contracting HIV, contracting syphilis what they talk, and getting pregnant to a girl child.

**I:**  Does someone get syphilis if he is involved in such things?

**R:** Eeh!! even a girl child gets pregnant.

**I:**  So, sexual acts and promiscuity, you said even in your community you have not heard about such things?

**R:**  Children get pregnant.

**I:** You know we are discussing this ting in young children.

**R:** They get pregnant when they are 16 years, sometimes they deliver. I had my girl child she had finished P7 the boy stole her from school by the way I came here and reported. was stolen from school and she got lost I searched, went to the subcounty Rugando I made a report investigated but the child got lost, she was 14 years… OC said the child is old and she going to a shame you, he said if she still loves her boy, leave her, to get married because she has spent a week already, you can’t bring her back home and she is a woman she does not want school, she went when she was 14 years.

**I:** So the OC said the girl is old?

**R:** When OC saw the physical appearance, he said she is old and she has tasted on the man, so if you take her back home she will shame you from there when she delivers from home. So that’s what he was meaning, he said that if you see you are able to take her back to school do it and if she does not want leave her to stay with her man and gets married. but even she came back because of the situation, because she was still a child she had got married in a family of drunkards, I told them face it, when months reach bring her to me. They sent my child, she delivered from here Kinoni.

**I:** How old was the man who impregnated her?

**R:**  I think he was also young. He was a bit older than her, he might have been 16 or 17 years, but it affected her, she can’t take care of children, so the children grew up from home. Because of alcohol so what did he benefit. she can’t do anything , she is still young, so things disturbed her.

**I:** Is there any child in your community who was involved in sexual acts?

**R:** I have not heard it in our community or in the nearby communities to say they have raped a child. but I hear them from the radio.

**I:** Have you heard of these issues of sexual acts where the child goes beyond, starts watching adult films, or sometimes when girls are walking they are touched on their sensitive parts.

**R:** They don’t sleep with these girls, they rape them, and sometimes they happen but now you see, you don’t know who has been seeing the child going, when it gets night you go to your home, what happens some where you don’t know. When you have grown, you see children and wonder but is not yours and the situation of these days when you talk about someone they take it personal ,children can be doing them at night, or us we are already old we can be in our homes.

**I:** You be in your homes?

**R:** Yes but the young children do those things. That they like to do them, you see young children, so when the child is in such a state, if you are the parent you see and wonder but if you are not related to the child, you don’t talk about it. You see they happen, you see the young children.

**I:** How do they conduct themselves?

**R:**  There are boys who have sex with young children.

**I:** Are the boys old?

**R:** They are somehow old, but when they are not men but when they have young girls.

**I:** Even he is passed 18 years but should not be marrying someone who is 14 years.

**R:** That does not exist, but now like mine I reported and money was extorted from me, that they go to Ntungamo where she was married and they arrest him. it comes from their peers, now if this is a neighbor, when a boy comes, he goes through this one (neighbor), and takes her for him, so even me when I found they had arrested him, so my girl never used to move. When she finished p7, her head was not good, I wanted to take her to a vocational, now when she was studying, when it was evening, I waited for her in vain. I rag the one who was teaching her, said my girl does not normally come late, she comes on time, now she is not yet here, where is she? That the girl left long time ago, I don’t see her! That she came. I tried to investigate from her friends, th second thing I don’t see her, I started looking from relatives, that my child is where and the one who had stolen her was hiding, but I kept quiet until I investigated where I wanted. The other boy took her and introduced her, took her and married her. They see the child is going to be well or to go in the right path, so the other ones made a deal to give her a man because they know these have failed.

**I:** When you reported at the police, did they test the man to know his status?

**R:** They never tested, but when I brought her back I brought her.

**I:** She was pregnant?

**R:** No, because she was a child, we brought her at the sub county they tested, she was ok.

**I:** But the man did not test?

**R:** The man disappeared and went.

**I:** In your thinking do you think if children involve themselves in sexual acts they get HIV?

**R:** So when they sleep with whoever they meet, how will they know if they are sick or not? but we would work together we first test as we are being told on radio, you know how the other one is standing, then you continue with your neighbor.

**I:** Have you met this situation in your community where a child has been involved in sexual acts and got infected of HIV?

**R:** Now the issue of HIV when a person falls sick you can’t know, now that’s why we can’t know it’s so and so. I think they can be there sick, but you can’t know it’s so because you see a person walking but you don’t know. But when you are asking them are you sick go and test,

**I:** You had told me that in your community you have not heard about such things, but you have said you think they might be there, according to the situation of the child, so do you think they are increasing?

**R:** I don’t know I can’t lie to you, because it’s like you have not caught them, but children of these days you counsel but the refuse to understand, even me I don’t want them involved in sexual acts when they are still young, but when the child gets involved in sexual acts where do you put it, they don’t miss, but as a parent if they are living on the village then you know they do them and when still young, but you can’t catch them, and say so does it.

**I:** Now in your community what is being done so that children are not involved in sexual acts?

**R:** May be sometimes they bring services in churches saying they help children. children who have AIDS they give them help and the second thing in churches they see people who cant help themselves, they help their children, like these support groups, there are many children so when they come from churches they keep giving them help but its not for everybody.

**I:** But. So that children don’t get involved in such things?

**R:** Me I have not seen people coming in this village gathering the youths like now they are in holidays, to see they are teaching, me I have not seen them. we even have VHTs but I have not seen them having time to sit down children to teach them to see what is moving forward.

**I:** Now you like a parent what measures have you put to make sure you protect your children from getting involved in such?

**R:** So you tell the child because for us parents we are to talk say my child see you are still young keep yourself from sexual acts, wait and first grow good things are a head, don’t run with so and so to spoil your peace and your youthfulness and when you feel not well in health go to the hospital there are services go and test to see your status. We also come this way to test to see our status. When you are a young child, you decide to settle if you have been protecting and yearning for to keep your respect. for us we talk but to put it into action is for our children. When they listen they come out but if they don’t they don’t come out.

**I:** Thank you madam for your time and accepting to discuss with us. is there anything you want to ask?

**R:** Now for you are there, this illness comes to us and we having to do about it now if someone is sick and wants and wants the services how does he get it and if he wants more information about HIV where does he get it?

Thank you

**Translation 2**

**I:** We are going to be discussing as parents how we can stop children to be involved in sexual acts as a measure to prevent the spread of HIV. Have you ever heard using a child in sexual acts?

**R:** Yes.

**I:** How do you understand it?

**R:** Having sex with children.

**I:** How do you know they are used without knowing?

**R:** Using a child in sexual acts to meet the child and lure it and rape, you meet the child sometimes you do something, you hold her by force and rape.

**I:** Have you heard about it?

**R:** Yes I have heard it

**I:** Has it happened in your village?

**R:** In our village it has ever happened. A man lured a child and was an uncle he slept with it, in our village it has ever happened..

**I:** You heard about that one only or there are others?

**R:** Others we hear them in the village not one by one we have always heard them happening.

**I:** That uncle who raped the child, did you hear about it or you were among the people eho arrested him?

**R:** Its not that we were there, but we heard from the village

**I:** So, what did they do to him?

**R:** They arrested him and put him in prison, but later released him.

**I:** Who caught them?

**R:** They were caught by the neighbor.

**I:** What did the village people do?

**R:** When they caught them, people gathered and called police had arrived and he was taken to police.

**I:** So in your thinking do think using children in sexual acts can cause them to get HIV?

R: Now if the person sleeps without a protective, the child will get HIV and other sexually transmitted diseases.

**I:** Now did they test that man who used the child in sexual acts to know his status?

**R:** They brought the child here at the hospital tested her and found nothing had happened, she was tested two times. They tested after 3 months they found she had no infection.

**I:** Did they test the man?

**R:** The man was not tested because they reached taking him to prison but the child they kept bringing her here, testing her. The child was found not to have any infection.

**I:** Do you think using children in sexual acts is there in your village?

R: We can’t say it’s not there because I saw it somewhere that one who did it they found his head was not well but we protect our children so much because boys are many looking for girls even when they find a young girl wants to defile her.

**I:** Using children in sexual acts means what?

**R:** Having sex with a child.

**I:** There is using children in sexual acts, there is mistreating the children, sometimes they watch their parents when they are sleeping at night in one, children and parents sharing it, or watching adult films. You find they touch the child, do you think in your community they are there?

**R:** You find children watching films which they are not supposed to be watched by children, but we decry that. Now when you are watching a film, you see something that is not supposed to be seen by the child, you remove it fast or you chase the children when you are going to watch such.

**I:** Now what do you do in your community so that children are not used in sexual acts?

**R:** Trainings, VHTs come and teach us now like us women, we are supposed to see children sleep alone. They told us how we are supposed to train girl children, the child is supposed to be counseled how to walk, how she will meet boys and distance herself from them.

**I:** Those are VHTs, what about LC1 chairman?

**R:** Now like chairmen, when you have brought a case to them, like that, they sit you down and talk to us but if it has not happened you can’t find them gathering us to teach us on such. Only bringing them such a case, they say protect your selves, tell your children, but if you have not brought it, I haven’t seen them collecting us to teach us such things.

**I:** What did police do to that man who slept with the girl?

**R:** Because he was related, when police arrested him,, they went there and punished him, but later they sorted the issues from home, but for police it ended there.

**I:** Now what has police done to prevent children from sexual acts?

**R:** When it happens, they arrest him, punish him, others learn from him, even you the parent you follow up the case , but when you leave and you don’t follow the case, police ignores because the parent who has faced such is the one who follows the case.

**I:** What has religious sects done to prevent children being involved in sexual acts?

**R:** When we are in churches, trainings of mothers come, they sit us and teach us how we are supposed to be conducting ourselves in our homes, how we are supposed to take care of our children.

**I:** What else has religious leaders done?

**R:** When the preacher is preaching, he brings in such message about young children, those who are there they hear it, they normally teach on such things.

**I:** You said you have a child of 11 years?

**R:** Yes

**I:** Is it a girl or boy?

**R:** The elder is a boy.

So what have you done/ you know boys also are used in sexual acts?

**R:** You teach the child, you tell him, that a person should not call you and put you in bad acts, or it it’s a girl you tell her if you meet a boy like this and this, or if you see things that are not good, tell me don’t fear me, you say I have seen this or that has told me this and that.

**I:** The one who tell her that they are to use her in sexual acts?

**R:** Now you see our children from the village go to gather fire wood, you tell her that you child, that in that place here are shepherds…when he calls you shout, run because he may rape you, kills you and throws you.

**I:** Apart from talking, are there other things you do to stop her from getting into sexual acts/

**R:** You can show the child that you like what they have asked you, you give it to the child so that they don’t met someone to give to them when you have never given it to her. When you call the child that come I buy you erindanzi,and it responds my mother will buy it for me, , you find you can’t try to lure herbecause the parent can give her all.

**I:** So do you know any case where a child was involved in such things and got HIV?

**R:** No

**I:** Now on what we have talked today, do you have anything you want me to explain for you?

**R:** Now am asking, when your child has been raped, you go to police you find they have released the person, is there another way we can use so that that person is heavily punished?

**I:** Is there another question?

**R:** You can continue to guide me how we can protect our children like us parents we want you add on that?

**Thank you.**

**Translation 3**

**I**: Have you heard involving children in sexual acts?

**R:** Yes I have heard it.

**I:** Tell me what you know about it?

**R:** Using children in sexual acts I say that children would not have reached the age of getting involved in such acts.

**I:** Which acts?

**R:** Sexual acts.

**I:** What do you think is involved in sexual acts.

**R:**  To find the child has men before the age.

**I:** Now men and children doing what?

**R:** Having sex.

**I:** Now when you say sex what do you mean?

**R:** I know things of home and when they have not reached the age of making a home.

**I:** For you where did you hear things concerning sexual acts?

**R:** Those things are common in villages.

**I:** Now like in your community where you live to find children being used in sexual acts are they there?

**I:**  They are there.

**R:** If we count out of ten how many people do you find using children in sexual acts?

**I:** Like 6 or 7 out of 10.

**R:** So you see how many often happen ,in your community is there a way you know it happens in children?

**R:** They are there but you can’t know is from here or there but you see them walking.

**I:** So being used in such things?

**R:**  Often times parents don’t mind about their children you find the child is being sent when it is passed time, or you find the child he/she is staying with is not catered for, or mistreating the child, you beat the child, you find the child has gone, somewhere and men start luring her to use her.

**I:** They use her,

**R:** But it comes from carelessness of the parent or the person taking care of the child.

**I:** So in your village have you heard a child being sexually used?

**R:** It happened sometime back.

**I:** How long?

**R: A year**

**I:** What happened?

**R:** The child had gone to un tether the goat, went when it was passed time, when she reached, she met people, she did not know who used her.

**I:** How old was the child?

**R:** She was 13 years old.

**I:** People got to know?

**R:** They tried investigating, they failed, they did not know, the child may have been knowing them and had been having connections with them but before they got involved in such. They tried to interrogate the child but she refused to reveal.

**I:** Now what did they do to the child when that happened?

**R:** They took the child to the hospital, tested her, found her infected, it seems the child was given medicine.

**I:** Now in your thinking, when children are involved in sexual acts doe it bring them HIV?

**R:** Yes

**I:** Why?

**R:** The person he uses for work doesn’t know where he comes from, only comes to rape, he has his motives as a person.

**I:** Do you think using children in sexual acts in your community are there?

**R:** Yes there are there.

**I:** Why do you think they are their?

**R:** Depending on how I see raping children under age.

**I:** Is there anything being done in this village so that children are not involved in sexual acts?

R: You go to see the child is pregnant, you as a parent you sort yourself to look for the person who impregnated her, police doesn’t mind when those are over, after the child has delivered they bring the person who did it when he reaches there, he is made to write an agreement, he accepts to do them, but later he disappears. After him disappearing, when you keep going there, they say they are tired of you. We are fed up of your things, go and sort your selves it’s you who knows.

**I:** Is there a way churches have been involved in protecting children against sexual acts?

**R:** I haven’t seen that, we go to church and pray then go home.

**I:** So we have seen what happens and that law makers don’t do anything, so you as a parent what have you done to protect the children from such a things?

**R:** The children I stay with, I don’t allow them to go visiting because visiting results in to such things.

**I:** Is there anything more you have done so that the child is not involved in such?

**R:** I talk to the child and I explain how they go but again in villages there is child you find is staying with the step mother you find they are mistreating him/her, they are the ones who have got such problems. Or you find the child grew up with bad manners, when the parent cannot manage her/him, some time we blame parents, but there is a child who has grown with a hard head, you talk but does not want to listen.

**I:** Some times when we talk about sex, you have talked or children raped, touching children in sensitive parts, children watching adult films, or children watching parents what they do. Now for you is there a way you have put so that you protect children against such things?

**R:** Now these phones we have, the child can steal it from you and you find is watching from there.

**I:** Those are the questions I had for you, do you have anything you wanted to add concerning such a things .

**R:** The question I have concerning from what we have discussed, if some one has a child, has tried to report to get assistance to those children who had delivered , what advice do you give so that that child gets some assistance?

**Translation 4**

I: As I had briefed you, thank you for accepting to have a discussion with us today. We’re going to discuss on involving children in sexual acts. We would like to see what you have gone through as parents and what measures have been put up to avoid such occurrences.

None of your answers will be regarded wrong, but you will share with me what you think about it, you’re the first person that am having a discussion with today.

I: Have you heard of using children sexually?

R: Yes, I have heard about it

I: What did you hear about it, tell me about it?

R: You hear of rumors, can you see that person by yourself

I: What do you know it is exactly?

R: I know it is sleeping with a man

I: Has it happened in your village?

R: Yes it has happened

I: How many times have you heard about it

R: Many times

I: For a few days past and the last years, when has it happened most?

R: It happened most on Easter

I: Can they know someone who has done it?

R: Yes they will be knowing the person and they punish him

I: Who caught them or who knew about it?

R: You won’t be knowing who caught them, you hear about in rumors

I: The village gets involved?

R: Ehh, of course it gets involved because it will have become like chaos

I: An old person who did this act, what did they do to him?

R: To a child they put him in jail, but when they are all old they are not jailed

I: They leave them and handle them in which way?

R: If it’s a child they put them in jail

R: They punish him from the village, they get together and punish them

I: What are some of those punishments like when they are age mates?

R: Sometimes they first take them for HIV testing, when they are not positive they are canned.

I: Do you think sexual acts can lead a child to contract HIV?

R: Yes

I: If it has happened in this village, is there any one amongst those who involved themselves in these sexual acts that has HIV?

R: Yes, it happens

I: Tell me about it and I see

R: In our village, we have not had that case and if at all it happens, they secretly go for testing

I: Has it happened elsewhere?

R: I always hear it on radios that a person meets with the other and infects her with HIV, but you can’t take it serious

I: In your village, you have not heard that some of those who involved themselves in a sexual act one had HIV and the other did not have it?

R: No

I: Do you think children find having involved themselves in these sexual acts?

R: Yes, it has to happen

I: Why do you think it must happen?

R: I observe these children, because even if you’re a parent you will have seen this child already, you look at him and tell who he is.

I: How do you do handle some of those acts that you’ll have observed as parents?

R: As a parent you council him, tell a child to control him/herself and after some time you again council him and the Lord does his part.

I: That’s how you have been able to protect yourselves as parents?

R: Yes

I: Are there other people that help you in that?

R: Yes

I: Who are they?

R: Political leaders, friends, and through sharing with friends you get relieved

I: Does the local council provide some help in this?

R: Yes the local council helps us, whenever the child becomes unruly and you report him to the LC’s, he changes abit

I: Do you normally have workshops concerning this issue in your village?

R: Yes, people come in our village teaching

I: Have you heard people teaching about sexuality?

R: Yes teaching about how to protect ourselves from diseases

I: How do they handle it?

R: They come and teach about sexuality, blood

I: Do they teach all of you or they only teach children?

R: Yes

I: Does the police get involved sometimes?

R: Yes they come when things get worse, do you think they just come when things have not become worse

I: So, for them they don’t come to teach

R: No

I: Do the churches teach about sexuality?

R: They only teach what they always teach in churches

I: Do you have any other method that you use to prevent children from getting involved in sexuality other than the ones you had earlier told us?

R: At times you tell him to avoid bad companies, you can’t restrict him from being with other people but even those companies can still bring him problems and so you keep advising him as a parent.

I: How do you handle the situation when you are not at home?

R: I won’t be there they’ll be, and I have three children, one boy and two girls, my children I gave them to the lord, because if it wasn’t for the lord then I wouldn’t manage, and they have reached adolescence, the older daughter is in senior one, and that is adolescence, but most importantly I pray that God hears me because even if I talk I still leave them alone and go, and I come late from Buteraniro after loading beans on a truck and reaching home is at 9:00 or 10:00PM, so by that time can I know what that child has been doing? But I think the lord who gave me these children and took away their father, am moving up and down looking for their school fees, let God reign, for me what can I do, because even if you talk, you are there or not if a child has no self control you can’t manage, even if you say that you’re controlling them all the time, but for me I can i say that I have control over them?

I: Do your neighbours help you sometimes?

R: My neighbours are my in-laws and my mother-in-law

I: Do they help you to control your children?

R: Yes I also tell them to be in charge of my children in my absence and my mother-in-law, but can old woman keep coming from her house to our house to check on the children? But for me it is only God’s grace that shall work for me because I can’t raise school fees if I do not work, how can I raise my family then? My children live by grace, With God as my helper my children will be successful and if he is not I will fail but most importantly I tell them.

I: How do you tell them?

R: I tell them bad manners first, I tell them to seek the lord for they are orphans and once you turn unto the lord he will make all things possible and at school your teacher is your immediate parent, so listen to your teachers and I still tell them about sexuality, that when you involve yourself in sexuality you contract diseases, you can be impregnated, things of that kind, control themselves, also teach them as a parent but the rest leave it unto the lord. Do I have to wake up every morning talking the same thing? Only God will see me through; me and my family because I can’t make it alone because I travel a lot only believe in God

I: Thank you for discussing with me, those are a few questions that I had for you may be if there is anything else that you would like to share with me about sexuality amongst children or any other additional information as a person that you would want to add that hurts you about such.

R: What hurts me is looking for school fees for my children, school fees for my children is what is on my mind because I now have the capacity to get it and the rest God will take care of them and give them to me. When my husband died I tested positive and even before he died like in that week we had tested, he is the one who first tested and told me that he was positive and I also got tested on Monday, first and second time testing my results were still negative but when I tested the third time I found I was positive. In that week he had taken tablets for like three times and me like two times but in just one week he fell sick and was taken to itojo hospital but immediately I came back with a dead body. He did not take long, but it showed he had been with the virus for some time but he was still healthy that I did not understand him, I also did not care because of my business and things of that kind like what you’re saying and all your time is set for business and forget about other things and so he died like that. After he died I kept on taking medication and it is four years since he left and four years on medication, I have also made four years and still living in fear am not yet used, but I have not told my children that am HIV positive, I will tell them but I have not told them. Because they are still young may be the one in senior one is getting old since she has started menstruation but I have not shared it with them and I keep it where they can’t find it.

Thank you.

**Translation 5**

I: Thank you for coming. As I had earlier briefed you we shall be discussing on the challenges and parents’ actions towards child protection against sexuality and contracting HIV/AIDS.

As I have told you we’re now working in rwampara district and therefore you can share with me about that issue though I will be recording your voice to avoid taking much of your time.

Have you heard of using children sexually?

R: Yes

I: You have heard about it, what does it mean?

R: Using children sexually usually comes when the parents mistreat them, does not take care of their children, those who live with their step mothers and mistreats them, denying them food and the child runs to where she can get like what to eat, or some money to buy yellow bananas and the child ends up being used sexually by men, not out of her will but because of saving herself of hunger but not knowing the end result.

I: So a child who is mistreated at home can lead her to getting involved in sexuality issues?

R: Can be misled when in actual sense wouldn’t have been misled

I: Have you heard of this happening in your village?

R: Even me myself I wasn’t supposed to get married soon because we were not poor but because of our step mother mistreating us, our father was rich but she could deny us food and lock us inside the gate, they had a hotel and lodges but would find us eating bones and those who could come in visiting the toilet had a chance to use us sexually. As step mother is locking us not to have food, when this person comes in and calls me, I would be rushing to have sex with him so that he could leave me with some money and we ended up in that mess because of hunger.

I: She would lock you inside the house to deny you food and would not give it to you at all?

R: She would not but rather would receive people disguising as customers yet they had come for us

I: Where were you living by that time?

R: I was living with my dad who was a principal of a school but we would stay at home with our step mother

I: Was it this village?

R: Exactly here in Buteraniro

I: Now that you are old and have had your own children, have you seen these things happening?

R: Yes; and very much

I: How many people have had this experience?

R: Like three people

I: Like in what period of time?

R: Last year two people were netted and in the year 2010, a man who was using his biological daughter was also jailed.

I: Has it happened in this year?

R: No

I: Who caught them then?

R: They were caught by a neighbor, who is like an in law

I: How did the whole village react towards that?

R: That person was taken to prison but because it was from one family nobody followed up the case

I: What did they do to the child?

R: The child became unruly and the mother went away/divorced

I: Did she go with the child?

R: Yes

I: And what about last year’s incidence, how did it go?

R: That girl was still young not work in bars but because of the conditions I had already told you, forced her to start working in bars but people kept looking at her as achild and when someone new comes to work in a bar every body will be starring at her and because may be she was timid, one day she fell sick and when she came here to visit a clinic, she became weak and lost strength from that playground, people defiled her from there and she even died, on reaching here she died.

I: They did not know them?

R: No

I: What about those whom they touch on their private parts, or they share the the same bedroom with their parents and their parents have sex in their presence, have you heard about those?

R: I have not heard about them

I: Do you think forceful involvement of children in sexuality can cause them to have HIV/AIDS

R: Very much, because they have not yet understood and even the understanding that we get as old people that when you have had a sexual urge you can use a condom, for them they will not have known it.

I: Are there people that you know who got involved in sexual acts and contracted HIV/AIDS?

R: Yes, even me the person I got may be because he knew I was suffering he came for me and the end result I discovered I was already positive and I had to leave him, the one I have today is a second husband

I: The child you told me who went with her mother, were they both tested?

R: The mother took the child before she was tested

I: So, the status of the child and the father was not known?

R: Yes

I: The girl child you told me about that was defiled, what was her status?

R: She was rushed in here for first aid but before she was tested, she passed away

I: So you believe that such acts are happening here in your village?

R: Yes, people are just jealous of others they only want to destroy other peoples’ children’s future

I: How do protect your children from getting involved in these acts?

R: We have a very big problem, but these boarding schools came to help us because when I child is in school she’s safe from these evil doers especially like us who are always in town and the children are home alone and the child has reached adolescence stage, they are only looking forward to destroying that child

I: How do you handle the situation when she comes back from a boarding school?

R: Make her busy with household chores so that she is with her parents most of the time and other tasks outside you send a boy child because these things normally happens on a girl child

I: What are some of those tasks that you do outside the home?

R: Like gathering firewood, fetching water especially where there is a distance to avoid her being attacked

I: Are there some authorities who could be behind these acts of sexuality on children?

R: They help us when it is already done like chairpersons they don’t come to check on us like how you have come to do this investigation, telling us to protect our children and put by laws restricting persons moving at night as it is by the local council ‘by-laws’

I: Have the churches offered any help?

R: They have tried

I: How has it been handled?

R: While they are preaching in church, a child who is not mistreated at home will pick the message such that even if she is convinced she won’t give in because she does not lack, so their hearts will follow the teachings they heard from the church

I: Do you have village committees?

R: Yes

I: Have they helped you?

R: They don’t normally make checkups in the village

I: Are there any other measures that you have put as parents to protect these children from falling into these acts or what should be done?

R: Talk to the child, because when he has known it when it is still early, she will grow up scared about, take care of her and provide for her needs not to envy her peers and lure her into that act

I: Thank you very much, that’s all I wanted to share with you but what do you think should be done to reduce these acts from your village?

R: I think if the VHT’S and chairpersons work hand in hand and make routine village check-ups and pass on information just like those who do family planning when they want to come they pass on information and we know it, they could pass on more information because we now have more ‘wild animals’ they don’t deserve to be called human beings.

They should increase on the punishments given to these ‘wild animals’ and children run away from them, children should not move outside at night

I: Thank you very much, may be if you could be having something else to add on what we have talked about

R: What I would like to add is that you should keep coming to teach us such that even us parents that have already contracted the virus, because we’re are already infected should not be jealous of these young children, these children are the future generation, it’s like when you cut a tree; it will die out but what sprouts will grow and so are the children; they are the ones who will help us and so parents should stop being malicious. Even compassion has helped much

I: How has it helped?

R: In case they realize that the child is under their care, they must make sure that that person is put to jail no matter what situation

I: And the child that has been involved in the act, how is she handled?

R: She’s taken for HIV/AIDS testing and the other is put in jail

I: Does compassion spare some time and get into the villages to do some sensitization?

R: They normally invite us and sensitize us from the project

I: Do they invite the whole community or only those whose children are under their care?

R: Only those whose children are under compassion, that’s why I was saying that VHT’S should work hand in hand with local council chairpersons because like now that you have invited us, it can be more helpful to them because you will been invited for a reason.

Thank you very much.

**Translation 6**

I: Like I had earlier briefed you, you are welcome and today we shall be sharing on the challenges and ways parents use to protect their children from sexual involvement as a way of HIV/AIDS prevention in Rwampara district.

I: Have you heard of children who are used sexually?

R: Yes, at times children copy it from their peers/friends

I: How do they copy them?

R: When they look at what their friends have done, they will also want to do it

I: How do you understand sexuality?

R: It is sexual intercourse

I: Will he be copying from a fellow peer or an old person?

R: He can see grownups doing it, with this modern generation you find people in action in presence of children especially on bars and the children also becomes anxious to do what these people were doing

I: But will you be knowing that what is being done is wrong?

R: Very wrong indeed

I: Have you heard of children/got information about children getting involved in sexuality?

R: One day I was at the hospital and some people brought in very young children that they were having sex, they were three boys who were having sex with one girl child

I: All the three boys using one girl child?

R: Yes all the three boys involving her in sexuality

I: They could have sex with her?

R: Yes they were plying sex with her and they a bit older than her

I: How old were they?

R: They were like eight, nine and ten years yet the girl was only three years old

I: What had they brought them for?

R: To test them and know their status

I: Did they bring the child the boys were using?

R: Yes, her father brought her first and after the boys were also brought in. one of the boys reported his friends

I: So they were all tested?

R: Yes they were all negative

I: Has it happened in this area of Bugamba?

R: Yes, very young boys copy their fathers

I: Will they be watching them?

R: I don’t know but there is when you hear them saying they are trying to do what their fathers do, I think somehow there is how they watch them

I: How many times have you seen these children doing this?

R: May be many who do defilement, some of those whom I know they defile them

I: How many cases of children do you think have been used sexually?

R: Like hmmmm five cases

I: All in this year or?

R: No, not in one year, like in the past years there is when they brought a child of like twelve years in 2017 and that girl was pregnant, it is a lot

I: Did they know who had impregnated her?

R: They did not know him

I: Didn’t she tell them?

R: Most children keep it a secret but I think her parents knew about it

I: But as for you, you don’t know whether they came to know him?

R: I don’t know how it ended

I: Do you know of any case similar to that of the other three children who had sexual intercourse with one girl. Who caught them that time?

R: When the father of the child brought her, it is the child who told them about those boys

I: Was there any community involvement on how to help them?

R: No, it was left for him alone

I: How?

R: They were forgiven, given strokes and since they were not positive they let them go. There are other two children who were defiled from mweeya and they were in great pain but they got medication and got better

I: Were they tested of HIV/AIDS?

R: Yes

I: Were they found negative or they were put on medication?

R: Okay, you see when they test negative they are given emergency pills

I: Were those who sexually harassed them put in prison?

R: Yes, he was imprisoned, he was one man on two girls

I: How old were those children?

R: One was like eight years and the other one six years old

I: How old was he?

R: He’s an old man of like thirty eight years

I: What did they do to him?

R: He was imprisoned

I: Can children involvement in sexuality expose them to contracting the virus?

R: Yes, they can contract it

I: In what ways?

R: Through sexual intercourse, using objects which can pierce, sharp objects which can make them ooze out blood, can make them contract the virus that cause HIV/AIDS

I: Is there any child in your village that have involved herself in sexuality and contracted the virus?

R: The one I know is not from my village but is from this sub county of Bugamba, an old man used her several times and the VHT is the one who brought her here and the child tested positive of HIV but the man who used her had recently died. The child started ARV’S at age twelve

I: How did the VHT come to know about this?

R: The child had taken some time walking abnormally and the VHT got concerned because she was even weak

I: So the VHT had to ask her what had went wrong?

R: Yes, she asked her and she told her then she was brought here

I: What kind of relationship did this child have with this man?

R: The man was from that village and child was living her grand mother

I: Is the child still on ARV’S?

R: Yes

I: What measures have this community put up to protect children from being abused sexually?

R: Aha! There is nothing to do, there are drunkards along the way, marijuana smokers, there are many people with different behaviours. And when a child is defiled, you may not know but when you get him or learn about it then you can report him to the authorities

I: What can the LC’S do upon such an incidence?

R: May be if you can contact the LC’S and they make sensitization upon that issue

I: But do they do it?

R: The LC is only there to resolve cases brought to him and forwards them

I: But don’t they inhibit this vice?

R: I don’t know

I: Is there any role that the health committee is playing to protect children from these vices?

R: May be it is there but am not sure about it

I: Is there any role played by police in this case?

R: The police is at the sub county

I: So the police has not been helping you at all?

R: Once you report a case to police it is resolved

I: Does the police prevent this act from happening?

R: No

I: Do the churches and mosques also have such cases?

R: May be because on Sunday they teach about those issues telling people to desist from such kind of behaviors

I: But do they only tell people that such act is bad or they don’t tell people to avoid it?

R: Yes

I: I see you have children, how do you handle them to prevent them from getting involved in sexuality?

R: The most important thing I do I tell my children to accept that someone sends her somewhere, someone to call her when am not with her and she goes there, but of course in my presence someone can send her for something and she brings it but when am not there she should not accept as she can meet those who defile children be taken to be sacrificed. As a parent you tell and when it happens it happens but having told the child

I: Those are a few things that I wanted us to share, may be if you could be having something to add or something else to share with me about sexuality

R: I would like to ask you, issues of sexuality in villages are increasing day , which way can you give us

I: Is there any other question?

R: Because these cases are on the increase, maybe it’s a curse? I don’t know but people are using children. Because a child was defiled by an uncle

I: Was she living with him?

R: The mother was going to weed millet and she left the child with him and on coming back she found she had been defiled

I: How old is her uncle?

R: He’s like fifteen years but he was put in jail, these things are continuing to happen, if you can offer us some help then help us

Ok thank you

**Translation 7**

I: So, you’re welcome……thank you for accepting to have a discussion with me.

R: Ok

I: As I had earlier briefed you, today we shall be discussing about sexuality amongst children

R: Hmm….

I: In our discussion we’re going to be talking about what you go through, what measures you have put up as parents to protect children from being used sexually such that they do not contract the virus. What do you do as parents such that your children protects themselves from contracting the virus. So, I will be asking you as you tell me what you know and how see these issues in your village.

R: Ok

I: Whatever you tell me is correct, it is how you think

R: Ok

I: So, Have you heard of using children sexually?

R: Ok

I: Tell me how you have heard about it

R: At times you find that there are some casual laborers’ in the village whom she’s very close to and ends up using her without your knowledge and at times the child will keep it as a secret and when she has reached eighteen years and you want to take her for testing you notice she’s already positive not knowing how she contracted the virus! And if it has already happened that the child has contracted the virus, as a parent you should not easily give up. You should consult the councilors for both your counseling and the child and she will live with the virus. But at times we get challenges when the child has started using any of the methods she is not used to, she begins to be stressed especially when she is at school, amongst her peers she starts to be depressed because she has the virus.

I: What do you understand when someone tells you about ‘sexuality amongst children? You as a person how do you understand it?

R: Sexuality in children means that may be a child is met along the way and forcefully engaged into sex or defiled

I: Are there incidences of that nature in your village?

R: Yes, it has happened. Even my elder daughter experienced it when she was three years old. A casual laborer who was working at my in laws’ home got used to my children. He had stayed there for three to four years but that act couldn’t be imagined, you couldn’t even think that would happen but it happened.

I: The child was three years old?

R: Yes three years and …….it was Saturday when it happened but I knew about it on Sunday evening and I brought her here on Monday that’s when they told me that she has been raped but a good chance she was not injured only what he had ejaculated in her is what was troubling her though it had not reached far, she did not bleed only I was given some tablets and we went back home because they told that the child was safe. Reaching home her father took her to mbarara hospital, and on reaching there she was admitted, we spent there two weeks and she was given all the necessary medication. She was tested and turned negative but we were given medicine of three types; tablets and syrup that was to be administered in same hour. I was to make sure that she takes the medicine at the exact prescribed time even if it meant I was going to the garden I would go with it. This lasted for one month and I was advised to take her for HIV testing when she makes five years. When she made five years, I took her back and she was still fine. And there after came in search

I: You mean IDRC?

R: Yes, they started testing in villages and people could win prizes and the first time I did not explain to them the exact issue, I just told them to test the child but they refused because had found I was negative and that automatically the child was safe also. I looked for ways to explain to them but I couldn’t. I went back, later came in a second search but the father had already passed away. I took her back for testing but they couldn’t and I kept feeling bad, later they came in the village to look for those who had refused to be tested and I secretly narrated to them what happened to her and accepted to test her. She tested negative and I had to give up on it because I now knew that she was fine may be if she could contract the virus through her negligence

When the VHT informed me that I come with the child, I brought her and that’s why she’s here. So, I have had this experience and I know much about it.

I: So did you test the casual laborer who did this injustice?

R: He went in hiding when he heard that I had taken the child in hospital. she had her uncle who was between sixteen and seventeen years. He told the girl that when she’s asked who did on her to say the name of her uncle. But I think it was God otherwise the boy was going to fall into a pit that was not meant for him. We found the doctor whom we found here was a relative and he told me not to rush things but rather go slow. He told me that I would rush to reporting a case to the boy’s father and he’s harmed yet he’s not the one that did not do it. Provided we have given the medication, after two to three days we will have known who did it. Coming from the hospital this casual laborer saw me and he knew I was coming from the hospital. He tried to understand whether we had known what had happened to the child and he found I was narrating to the grandmother what had happened to the child. On realizing that we had already known about it he entered into the house where he used to live and escaped from the back door. They put up a search but he was nowhere to be found. But it was found out that where he had worked before he had the same habit, he had defiled a child as well but when they knew about it, nobody took interest in the case and he had to search for a job elsewhere. A search was mounted on him up to now but he has not been found.

I: Are these injustices still happening in your village?

R: Yes, even up to now last year it happened on a neighbor I don’t know if you will be the one to share with the father of that child or it will be somebody else but there are the children. It was done by my in law called humprey. He’s positive and depressed he’s even on ARV’S, he defiled them but they came to know about it after some time, they never bled but they got him and beat him when they knew about it, he did not admit but because the children had grown up they reported him. He was taken to Kyamugooranyi government prison up to now he’s never come back

I: Were the children infected or not?

R: I don’t know about it but he was jailed and he’s not at home. Many have kept silent about it but if you were to bring all of them here you would hardly have where to sit. It normally happens but it is commonly done by casual laborers and those who are hopeless almost behaving in a lunatic way and is jealous of such a young child

I: Hmmm…So, that can make a child contract the virus?

R: True, it can make a child to contract the virus. Agh agh………

I: So, you don’t know whether these children are positive but the other one was put in jail while the first one ran away

R: Eeee….. I don’t know where the one who did it on my child went but the one who did it recently was put in Kyamugooranyi prison

I: So, it’s a lot that has been happening in your village?

R: Ummm….

I: Have your village come up with some measures to stop these acts from happening?

R: If it wasn’t for such meetings that you invite us in, there are no other services because you can’t share something that has happened before. Instead advice is given after an incident has happened. If at all before employing a casual laborer one would have to report him to the authorities and collect meaningful documents from him such vices wouldn’t be going on. In case it happens, it would be easy to trace where this person’s place of origin and is punished. But the one who used my child, the employer himself did not know his origin. And because he was working for my in law who is an uncle to my child the father of the child did not feel comfortable to put in prison his elder brother. Most importantly we asked for God’s help and the child was negative. He could not imprison his elder brother because he wasn’t the who had defiled the child

I: Ummm……

R: That calls for responsibility amongst the local councils and even us the employers. If you employ someone whom you don’t know his whereabouts, report him to the chairman and acquire his identification and you know that such a person is from a certain place. I live with my brother, I have his identification and when he came I took him for testing and I know his status. But the problem arises from the employers who just pick someone along the way and give him a job without knowing where he comes from, how he behaves and you cause problems to your neighbors

I: So, there is nothing that the LC’S have done to curb this vice?

R: No, he only tells you that if you have employed someone he should identify himself/documentation but won’t be interested in knowing whether you got the identification. He will not tell you to go and report the worker that you’ll have got

I: Are there some VHT’S or health committees that are helping you to stop this vice from happening?

R: Some of the selected VHT’S most of the time they organize us and advice us on how to handle our children and we have started to benefit from their services. When you are hard up you reach out for her and that’s why they are in charge, they organized us to come here and if it wasn’t for the VHT’S, then we wouldn’t have known about it.

I: Ohhh……Does the police do anything to stop these incidences from happening?

R: The police comes in when the case has already happened and in case you don’t have some money they end up not helping you, they tell you to buy medical form, take the child for medical checkup and to imprison someone they ask you to pay some money else they set him free

I: Has the church done anything to stop such acts from reaching onto young children?

R: When it is a holiday the church organizes youth services with ages twelve onwards and it teaches them about such issues giving them advice and therefore when you tell a child to go church she understands

I: As a parent, what have you done to prevent children from getting involved in sexuality?

R: That’s what I had earlier told you that you talk to them, give advice he will listen or else if he doesn’t want to listen you leave him but it doesn’t mean that you shall ignore completely, you keep talking to him as a parent and he can pick some sense out of it or otherwise

I: So, you talk to them?

R: As parents we talk to them but the rest is left to God

I: Hmmm….. all to God?

R: All to God

I: Are there any other measures that you have put as parents to prevent these vices from happening apart from talking to them, I don’t know?

R: It is important to advise them and show them that it is wrong, when someone calls you don’t get overjoyed, don’t show you’re willing and report it to me that someone was disturbing me. And as a responsible parent you take the initiative to confront that person and tell him to stop disturbing your child and in case you attempt to do it again, I will report you to the authorities he can get scared and the child is set free.

I: Eee…. Thank you that’s what I wanted to share with you. May be if you still had something else to tell me about sexuality in children

R: The most important thing that am requesting you ,do not stop from asking parents questions. Assist us to engage the government such that all children aged ten to eighteen years are taught and told that it is a bad act. As parents we talk to them but they feel what we’re telling them is not correct but follows her heart instead. But if the government organizes programs to talk to them in schools or you meet them from here and you teach them, you find like flavia has come all the way from mbarara university to care of my child, the child gets concerned. She will keep meditating about flavia’s love towards her to come all the way specifically for such a lesson and the child will know that it’s a wrong move. You will motivate her to be like you

I: Eeee….. I have understood. A child can easily understand someone whom she’s not used to.

R: More than her mum, eee….because she’s used to her mum and she thinks that’s how she is. That’s what we mostly need

I: So, such can guide children?

R: Very much, I can’t tell her myself. She’ll keep wondering how flavia went to university without HIV/AIDS, how did she make it? I have to emulate her

I: I think while we were growing up such things were there

R: Yes, it used to be like that, that’s why you used to hear of aunts of children but they are no longer there. It is everyone for himself everybody takes care of themselves

I: So, it is a parent to take care of her child?

R: Even an uncle cannot correct a child who is in wrong

I: What causes it?

R: If a biological parent can defile his own child, can her uncle advise her? If it can be done by her grandfather, can her uncle advise her? It is upon a parent to take care of their own children. Because for the past years correcting children was every parent’s responsibility even if the child wasn’t his he would punish that child and would know her wrong doing but today in case you make a mistake and punish someone’s children, you’re handed over to the sub county authorities. That’s why they go astray and end up contracting the virus and has increased multiplication of the virus. During those days correcting someone’s child was normal but if you did it today you would end up in courts of law and has led to increased to the nation’s deterioration

I: Ok thank you

R: Ok

**Translation 8**

I: Thank you for coming madam

R: Ok

I: As I had earlier briefed you in our discussion we shall be sharing on what parents go through in protecting their children against sexual abuse or getting involved in sexuality as a way of protecting them from contracting HIV/AIDS. Have you heard about a child who has been abused sexually?

R: No I have not heard about it

I: Do you understand when I talk of a child being used or abused sexually?

R: It is sexual immorality

I: Have you heard anything about that in your village

R: I heard about it once in some place far away

I: Where?

R: Some far away distance but in Bugamba

I: What did they tell you?

R: They were saying that a man had defiled a child, In fact I have seen that child she has come with her father. I think you will talk to her

R: I think she could have shared with my neighbor because there is someone else that I have come with

I: So, that’s what you heard?

R: Yes

I: Who did this evil unto her?

R: He was a relative

I: Have you heard about this evil somewhere else?

R: No, I’ve not heard about it elsewhere

I: Do these acts normally take place in your village?

R: No

I: Have you heard of a defilement case around your village even if it may have happened some time back you can tell me. Even if it was committed by only children or an old person before a child you still can tell me

R: No, I’ve not heard about it in our village

I: The one you were telling me about who sexually abused a child who netted him to the extent of being known?

R: I think it was her relatives because it is far from here. Am not sure of the place

I: What did they do to the person who defiled a child?

R: He was imprisoned

I: Do you think sexually abusing children can expose them to a risk of contracting HIV/AIDS or not?

R: Yes

I: In which way?

R: through sexually abusing them

I: Do you know of any child who has ever been sexually abused and tested positive to the virus?

R: No

I: You had earlier informed me that a certain man defiled a young child and was imprisoned, was he tested to know whether he was positive to the virus or not?

R: I think they had knew about it because we were told that he was positive

I: What was done to the child then?

R: I don’t understand those issues very well because there is some distance away from where I live

I: Basing on your village, do we have such acts of defilement or sexually abusing of children?

R: No, it’s not there

I: Why do you think it is not happening here?

R: I would have heard about it

I: What measures have been fronted by your community to prevent sexually abusing of children?

R: Taking care of your own child

I: Are there some measures that have been put up by the local councils to prevent sexual abuse of young children?

R: Local authorities keep telling us to protect our children?

I: How should you protect them?

R: They should be taken care of, should not send them outside late at night or during evening hours

I: Are there any other people in authority that you have seen talking about such issues?

R: Yes, chairperson LC1

I: Are there any other people apart from chairmen?

R: No

I: You had told me that you only have daughters and you’re now living with a boy who is your son in law, as a parent what measures have you put to prevent this child from being sexually abused? Because even if he is a boy child, he can also experience it

R: Yes it also happens

I: What measures have you put up to prevent your children from being sexually abused?

R: You talk to the child

I: What do you tell him?

R: That you should not involve yourself in things you don’t understand, constantly rebuke the child

I: As a parent and an old person, what advice would you give to other parents on how to advise their children such that their children do not have such experiences?

R: Children no longer heed to advice, they are always escaping and go where you don’t know

I: What other measures can we put forward to protect our children?

R: Introducing them to vaccines that inhibit the virus(the participant meant PREP and after mentioning it she laughed). What else can we do?

I: Are there any other measures that can be enforced to reduce sexual abuse of children?

R: Can it be reduced? Hmmm…… a girl child of this generation in adolescent stage is impossible to handle, hmm……. No

I: Why is it impossible to protect her?

R: They don’t listen because they want to be with their peers

I: What do you think can help her to settle down? To settle down and takes care of herself to avoid such evil experiences

R: What can we do to her? If you tell her and does not listen you give up on her

I: Do you see church leaders involved in the fight against defilement of young children?

R: Yes

I: How are they involved?

R: They mostly tell parents to take care of their children

I: How should they take care of them?

R: Take care of them

I: Do they sometimes talk to children and they listen

R: Yes, they talk to both parents and children when we are all in church

I: How does the local council authority advise you?

R: Sometimes they call for a meeting and share about it

I: Those are a few questions that I had for us to share today. Do you have any question for me before we end our discussion? Or else would you like to add something on the issues of using children sexually?

R: No, but also put some measures to vaccinate them

I: Do you mean to keep coming to vaccinate them?

R: Yes

I: Won’t it be harmful to their health?

R: What else can we do about it? May be just to kill someone who will have defiled the child

Ok thank you for sharing with me.

**Translation 9**

I: Once again you are welcome, as I had earlier briefed you we shall be discussing on those children who are sexually abused and I continue to welcome you in this discussion and thank you for your time. Your thoughts and whatever you tell me is very important for this discussion. As I’ve told you, the discussion is about children and measures that parents have put up to prevent children from getting involved in sexual elements and contracting HIV/AIDS while they still young. This discussion will last for at least twenty to thirty minutes. so, I encourage you to be patient as I ask you and request that you accept to record your voice and as you’re answering try to raise up your voice so that I can hear you. I promise you that this discussion remains confidential and therefore it remains between me and you. But try to keep whatever we shall discuss.

I: Have you heard about using children sexually?

R: Yes, I’ve heard about it

I: What was being said about it?

R: I heard of children who were like fourteen years old and most of them were living with their guardians, and those guardians involve them in such acts or when there is a casual laborer at home, you find he is engaging them sexually when parents don’t know anything about it and they end up getting problems

I: How do you understand it?

R: This is how I understand it, sometimes trying to deny or protect children if you’re a parent with a child you’re supposed to sit down and keep talking to your child especially when she is twelve or thirteen years. Those children will have understood especially girl child. Sit with her as a mother and keep advising her on the dangers of getting involved in sexual affairs, tell her that she will contract the virus that causes AIDS. Once you engage yourself in sexual immorality, you contract the virus or get un wanted pregnancies and get problems. Keep telling her to stop her from getting involved.

I: Where did you hear it took place?

R: In the centre, there used to be two neighbors and one neighbor kept using other neighbor’s child sexually. The child was twelve years and the man was like forty years and in the end this man had promised to give her four thousand but failed to keep the promise and the child reported the matter and when they took her for testing, her results turned out positive.

I: How many times had it happened?

R: They had done it for several times but would not speak about it because when they knew about it the child was already positive

I: So, they managed to know the person who defiled and the child?

R: Yes, even that man was imprisoned till today.

I: Who came to know about it first?

R: The child was staying with her grandmother, her uncles who are brothers to her mother did put him to jail.

I: Did the community get involved?

R: Yes, it got involved, their family opted to be given money and do away with the case but the whole community together with the chairmen couldn’t buy the idea and the man was first put in prison

I: So, next the man was put in prison and he is still there?

R: He’s still in prison

I: What about the child?

R: Her mother went with her to Kampala

I: Do you think that using children sexually exposes them to a risk of contracting the virus?

R: Yes, it can, because you are not sure of the status of this person that is using her, and remember the child is young and is not mindful of getting tested first and she is seduced by minor things. Even a sweet can make her accept to do it. So, it is a very bad thing that exposes children to the virus that causes HIV/AIDS

I: Did they come to know whether that this man was positive?

R: Yes

I: What was done to the child when they had noticed she was positive?

R: She was given ARV’S

I: Do you think this habit is still there in your village?

R: I think it is still there because we couldn’t imagine that man could do such a thing but when this happened I thought there could be others. Parents should engage ourselves in talking to our children about such issues

I: What community measures have been set up to protect children from being used sexually abused?

R: When this incident happened, the town mayor organized a meeting and he said, during this COVID period when children are not at school parents are supposed to be extra careful and keep guiding your child to avoid the same thing happening on your child. That you keep talking to your children as you rebuke them. A community meeting was held and everybody was told

I: What did the church talk about it?

R: Nothing

I: What about the health officials?

R: For them they talk about it everyday

I: What measures have you put to protect your children from being sexually abused?

R: I live with my children here in the centre where I have my business and I make sure that they are with me all the time, I must know where they are going in case they are going away. When am not here then they are with their father talking to them sometimes and at times they are with someone who rolls chapattis. At times I talk to this girl because she’s the old one. At times she is looking at those people in the bar but I tell her not go back to bars since this is a centre. These girls you see in bars end up in prostitution, sexual immorality and end up contracting HIV/AIDS they are impregnated and I discourage her from such bad companies and tell her that in case you join them you die soon before eighteen years and once you get pregnant before age you can’t manage to produce a child. You’ll have destroyed your future, would you be able to go school? I keep telling them about such issues. I restrict them from walking at night because she can be defiled along the way.

I: Have we left anything behind concerning sexual abuse amongst children?

R: I look at some parents who give too much freedom to their children and they are free to go wherever they want yet the child is only sixteen years and it is 8:00 pm and the girl is talking to boys along the way, the mother doesn’t know where she is and I keep wondering how that mother is going to raise up that child. Has she taught her anything and you find that it is a very bad practice.

Thank you for discussing with us.

**Translation 10**

I: As I had earlier briefed you, our discussion is about challenges and measures that parents have put up to protect their children from being used sexually. As one way of protecting their children from contracting HIV/AIDS. So, you will help me to answer these questions as you will have understood them.

I: How do you understand sexuality acts?

R: It’s like when a girl accepts to talk to a boy and they discuss like what boys and girls do, whether they agree or not and when the boy gets interested he marries you off; like that

I: If anyone told you about parents protecting their children from being sexually abused, what do you understand?

R: Explain the word sexuality first.

I: The child will be below fourteen years and is not old to be involved in sexual acts. It is like defiling children and it involves defilement, bad touches in a child’s private parts and exposing them to pornographic films, there is even homo sexuality and lesbianism and they all define child how children can be defiled. And we would like to understand whether as a parent you have heard about that.

R: I have not experienced it with my children but I have heard it somewhere else

I: So, that’s what we have come to understand how children can be sexually abused or defiled or being used sexually while they still minors. Have you heard about it in your village?

R: It happened and a man was imprisoned

I: What happened?

R: A man defiled a minor of thirteen years and the man was forty years and above and he was HIV positive and even when the child was tested, she also tested positive

I: Did she start taking ARV’S?

R: I don’t know

I: How did it come to be known that the child was defiled or has been defiled?

R: To me it was a hear say because it happened in another village

I: After this news, what did the community members do?

R: I don’t know but I hear the man was put in prison

I; When did it happen?

R: Less than a year

I: Are these kind of behaviors always happening here?

R: No, because I had not heard about it in our village

I: You told me that the man was put in prison but was this child introduced on ARV’S?

R: I don’t know anything about that

I: In your own view, do you now that defiling a child exposes her to a risk of contracting the virus?

R: Yes she can contract it because she’s not sure of the status of this person she has slept with

I: Lie I’ve explained to the different ways of sexual abuse in children, is there any that you have seen in your area happening?

R: Yes

I: What measures have your community put up to fight sexual abuse?

R: People are usually taught

I: Who teaches them?

R: Through listening to radios, radio talk shows

I: So, it is only done over the radios?

R: Parents should also teach our children and tell them to run away from men when they are still young

I: Does the LC committee in your village have the initiative to stop this bad habit?

R: May be when they are at school that’s when they are taught such

I: How about our churches, have they come out to criticize this bad act?

R: They teach about it during Sunday services at church

I: How do they teach?

R: Shall not engage themselves in sexual immorality when they are still young. An old man should not call a young girl and respond immediately as they are the champions of this evil act

I: As a parent with children between nine and fourteen years, what measures have you put forward to make sure that your child is not sexually abused?

R: We sit together and talk because I have two daughters and they are the old ones, the youngest is a boy. I talk to the girls advise them but you can’t tell what they can be thinking about. At times you rebuke her but still don’t listen

I: How will you be talking to her?

R: I tell her to run away from men and young boys because you are a young girl. Men will destroy your future, so settle and study. First study and get into such things

I: How does she avoid them then?

R: When a man calls her won’t she refuse?

I: Are there other things you do or behave at home such that children are well secured to avoid getting involved in such acts?

R: I visit them quite often or invite them to visit me

I: Where do they live?

R: They live with their relatives. I sent them there because their father passed on. But it near I see them everyday

I: They are not being disturbed where they are?

R: Yes

I: Do you have a health committee in your village, there is a time when they come to check on you sharing with you about such issues?

R: I have not seen them

I: Those are the questions I had for you today but I don’t know whether you have a question or any other issue to tell me or advice on how we can help children from being abused or getting involved in sexuality when they are still young

R: I would like to continue to advise my children not to engage themselves in such acts. How can I continue to give advice?

Ok thank you

**Translation 11**

I: We welcome you once again and thank you for accepting to share with us. So, as I had earlier briefed you, our discussion is going to be based on challenges and measures put up by parents to protect their children from being sexually abused as a way of protecting them from contracting HIV/AIDS. Our research is based in Rwampara. This research is being carried out in Rwampara district and whatever we’re going to be discussing will be rotating about that.

R: Hmmm…..

I: Have you heard about sexual abuse in children?

R: Yes, I’ve been hearing about it

I: You have been hearing about it, try to be audible. According to you what do you think it is?

R: Since quarantine started, this sexual abuse issue when children are no longer going to school and are at home you find a child is being defiled by her parent and the child is not sure about the status of that person that will have used her. And it becomes easy for her to contract the virus.

I: As you have explained to me, do you think that using children sexually comes as a result of them getting involved in sexuality?

R: It is about sexuality, sometimes a child is born with the virus and you find both parents are positive and they are on ARV’S or they are discordant couples and end up produce a child who is infected.

I: If you find that children have involved themselves in sexuality, how do you know that it is wrong or a child has involved herself in sexuality?

R: It becomes easy for you to know about it as a parent, a mother. But it is not easy for a father but if you’re free with your child, talking to her and the child is obedient it becomes easy for her to tell you everything.

I: So it depends on a child’s behavior and that of a parent?

R: How you relate with your child; she can be free to tell you even if she was forcefully raped from her way on the spring

I: Have you heard these experiences in your village?

R: Yes, it was April last year during lockdown

I: Ummm………

R: The man was a boda boda rider who had a wife and children but when a woman had come from the banana plantation, she found the man had packed the motorcycle and had defiled a child. The child was bleeding.

I: Was she his daughter?

R: Yes, a daughter, and we wondered what caused had it? He was put in prison and he has not come back

I: He has not come back?

R: Umm….if you can defile your daughter!!

I: Since the man was imprisoned, what was done to the child?

R: She was taken to the hospital, given medication and she became fine. But her father was not positive so she was not infected with the virus but he injured her.

I: Regarding your village, do you think it is much or less for example like last year, do such incidences normally take place or not?

R: These incidences are always happening but it has more than doubled during this lockdown

I: It has become too much?

R: Yes

I: Umm…what is the percentage of this incident happening in ten people?

R: It can’t be forty five percent, it exceeds this because ……

I: It goes up to what percentage lie in your village? And remember that is the only you knew about. Do you think there are many others that you have not known?

R: That is the one we got. But there is also where it is done secretly; may be she’s not injured and as a parent you don’t even know about it and the child keeps getting used, when she reaches fifteen you find that she’s pregnant

I: Umm….

R: Have you understood? Or else she contracts other diseases she does not know

I: Umm…. What of the other one you were telling me about, when the wife found him having done that, did the community try to get involved to express your concern and do something about it? Or else it was resolved as a family and took away the man?

I: No, what could we do as a community? When the woman saw the child had gone through this, she reported the matter to police and took away the man. Is it understood?

I: Eeee…..have you heard of other cases of sexual abuse of children in your village?

R: Yes, children have started getting married at early ages

I: Do they decide on their own or not?

R: How can they decide on their own? The Uganda we’re in today, every child needs money. They want quick money without working especially under this lockdown you could be telling a girl child to accompany you in going for weeding millet, cleans utensils when you wake up, sweeps the whole compound but because they have come back from school they are always in bad groups and it has become too much.

I: According to your thinking, when a child engages herself in such things or she is forced into sexuality; can it expose them to risk of contracting the virus?

R: Yes, it exposes them to contract the virus. Because you won’t know that am having the virus at that time before we have met in the hospital to receive ARV’S. Have you understood?

I: Umm….. so, now do you think that when a child engages herself in sexuality exposes her to a risk of contracting the virus?

R: Yes

I: Okay, so, you’ve told me that it is through sexual intercourse that she can contract the virus

R: Yes, it can expose her to contracting the virus or contract diseases that arise through sexual intercourse while still young. She won’t know the status of the person she’s going to engage herself with, how to protect herself, how to behave, and the methods to follow. She still thinks as a child. She won’t think about the future results that she can contract the virus or she’s impregnated

I: Umm…. Do you know of a child who engaged herself in sexuality and ended up contracting the virus?

R: Yes

I: You have heard about it, that a child who engaged herself in such acts contracted the virus?

R: No, I won’t tell you that I know about it, but according to how we sense issues as parents, a child who is involved in such acts is at risk of contracting the virus. She may not talk about it that she has contracted the virus, is infected with syphilis, keep dodging her parents, but will learn about it in future where it all happened from. But as a parent, it is won’t be easy for you to know about it

I: You earlier had told me that when children were sent home and lockdown came in, these issues became rampant where children are sexually abused.

R: Yes

I: Why do you have a feeling that it is on a rise?

R: You see, before lockdown, children have been coming for holidays and reporting back to school and you would hardly find time to discuss with them at home, when they would have come for holidays, they would still go for holiday coaching and you find that all the time they are fixed at school. She could not meet with people along the roads, she won’t be having that time share with you what she will have met along the way. But this lockdown has given them much time

I: As a person, what shows that these things are happening, yes, time is available but as for you how do you know it happening in village?

R: Why I think it is happening, I get my radio and listen to the news, I heard of someone who was doing her senior four final exams and gave birth and when you hear about that you think of the one you have in your house because since this one was doing her senior four exams she was in lockdown after school and your child is still under lockdown and has not gone back to school, that means she has to go back when schools re-open and on reaching to school you hear she has stomachache and things of that sort and reaching the hospital you’re told that she’s HIV/AIDS positive.

I: So, you’re thinking like that because children have spent long time at home and these acts must be going on?

R: It must be going on because that’s it

I: But you have not seen this incident by yourself or something else happening in your village which is not the one you talked to me about? Because what am about to tell you is adding on sexual abuse or sexuality in children. Are there sometimes when you find there are bad touches in private parts or breasts or you find they are watching pornographic videos

R: Umm….

I: Umm… such issues. Have you experienced them in your village?

R: No

I: Umm….okay. so, what is being done in your village, like leaders protecting children from having such bad experiences of being used sexually? Is there anything that the LC’S have done?

R: Eee.. the LC’S have done a lot for us. We have mayors that are fighting this but they are trying as leaders

I: And what do they do?

R: Just like yesterday, we were on burial ceremony and the mayor told us; that you parents should forgive me but if am passing around town and I find two or three children standing in town , I will pick them with a car and put them in jail. Because they don’t have what to do, they don’t want to help their parents but when they gather together they are in rumors, they start planning how to go and steal sugar cane, break into people’s homes because they have stayed long at home and they want money

I: Ummm…. So, they have been talking about this issue of children getting involved in sexuality?

R: Eee… even concerning sexuality, when boys and girls come together, you find them in close contact and they spend most of the time together and thinking to go home it is coming to 07:30 PM in the evening and every one ends up getting what he wants. Have you understood that?

I: Umm…

R: So, that’s what he meant by saying that when he finds two or three children standing by the road in the trading centre, your parent has no shop or bar in the centre, I will take him to police and it will be your parents to bail you out. I asked them whether they can’t go weeding. Can’t you use your child to help you in weeding? And while he was telling us , we realized he is right. It is upon us parents to see how to handle our children and get close to our children, advise them and tell them that since you are not in school, this is how they plant cassava.

I: Umm… I have understood?

R: Umm…

I: Has the church helped you in those issues or not? Have they shed some light about it?

R: Yes, since they opened churches, they have taught us, they teach children on how to fear the lord and they remain hopeful that even such does what? Also happens

I: Okay, so, you as a parent you have your own children either in that age group or above. As a parent, what measures have put up? To see that your children are not sexually abused or does not get involved

R: That is God’s plan. Because I can’t tell you that I can control my children to say that I can protect her from being a nuisance, won’t involve himself in conning girls, opening of schools. We have been protecting them from home because it could have been a chance that it is your child that didn’t get pregnant or has come from lockdown without any problems. But when we take them back to school, they still can get problems from school. So, as a parent I will be having a thought that am infected, I produced my child not infected and therefore I won’t like her to do what? Get sick

I: As for you, you’ve told me that you have been protecting them from home, what measures have you been imploring?

R: I have left it to teachers

I: Left it to teachers!! Before you left them to teachers, how have you been protecting them?

R: I would sit at home, we share and tell them that my children when you join bad groups of people who are not your age mates, you will contract diseases and after having our super I would send them for a tin of my medicine and ask them, have you seen how big it is? I show them that what am telling you tomorrow will you shall also swallow it! You speak to her making her to hate this medicine that you are taking so that she keeps fearing it. At times to find that she contracts a disease she will have done what? Will have grown up and if she’s to give birth she will have given birth or finish her studies. Eeee

I: Umm…. Okay so as you’re sharing with them you would teach them the dangers involved and see how to protect themselves and how to take care of themselves

R: Umm….

I: So, those are a few things that I had for you; I don’t know whether you have something you would like to add for me in issues related with children being used sexually.

R: For me I had wanted, all those questions you have asked me and given you my responses, how best can I continue to do it such that I live well with my child?

I: We shall discuss that later. Do you have any other questions before we finish?

R: No

I: Umm.. thank you for sharing with us, that’s all we had for you today

**Translation 12**

I: Thanks for accepting to share with us. Though you are not feeling well but you shall bear with us you will be attended to after this discussion.

P: Ok

I: Your views in this discussion are more important a for this study. The discussion is about challenges children face and measures parents have put to protect children from being sexually abused what many say it is defilement. Defiling children does not mean entering their private parts only. But even if you touched her breasts then you will have defiled her or even if you made that child to watch pornographic films before the required age, that also is amongst what we term as child sexual abuse. We would like to understand what measures parents have put in place to protect their children from involving themselves in these things I’ve just explained to you. Our discussion is going to last for about thirty minutes and we shall be recording our voices in this discussion and this discussion remains a secret. In case you have a question, you ask me from where we will have reached in the discussion. The first question says; have you heard about sexual abuse amongst children?

P: Yes, I normally hear those stories on radios but I have not witnessed it by myself

I: Where did you hear it that it happened?

P: I can remember about it but I heard about it of recent from this village

I: Of recent?

P: Yes

I: So, like you have heard about it, how do you understand sexuality?

P: I think the one who can do such a thing is lunatic. That’s how I think for sure because someone shouldn’t do such thing

I: As you heard it, as you think, what it means, do you think the person who does it is abnormal. Now how do you understand sexual abuse?

P: Do you mean a child that has been defiled?

I: Yes, how you can know that a child has been defiled or what will be her state after she has been defiled?

P: That can be brought up by a parent who knows how her child is feeling for the day or realizes that the child’s walking abnormally and finds out why. As a parent you try hard to understand your child and tell what could have happened to her.

I: You told me you had heard about it?

P: I hear about it

I: Where did you hear about it?

P: Over the radio

I: How about in your village or else while you’re going to church or to the market?

P: I have not heard about in our village

I: What about here in mwizi?

P: No

I: So, it has not happened in your village?

P: Yes

I: This that you have been listening to over the radio which is not in your area, how many times have you heard about it?

P: I’ve heard it over the radio for like three times

I: According to how you have been listening over the radio, would they be reporting to have managed to nab the one who will have used that child?

P: Yes there is someone whom they got but I didn’t follow the conversation very much. But it happened and I heard about it

I: So, the child who was defiled knew this person who did it on her?

P: Yes

I: According to how you heard the conversation, did you feel any concern from the community about that issue?

P: I did not understand about this but that’s how I heard it?

I: So you did not understand what followed next and what happened to the child?

P: Yes

I: According to your understanding and how you think using children sexually can expose them to a risk of contracting the virus that causes HIV/AIDS?

P: Very very much, I say very much because the child’s private parts are still small and therefore at a very high risk of contracting the virus

I: So, the risks are very high for this child to contract the virus?

P: Yes

I: How does it happen that a child can contract the virus after being sexually abused? According to the various ways through which the virus is spread

P: A child is forcefully penetrated into while still young and she even bleeds and she contracts the virus because it spreads very high through blood

I: So, the three times you’ve told me did you hear saying that all three children contracted the virus or did not?

P: No

I: So, you did not understand what followed next; that either the child got infected with the virus or the one who defiled had the virus?

P: Yes

I: In your own thinking, do you feel like the habit of using children is happening here in your village or it is not?

P: No, no one because am on LC committee of our village so I would be knowing about it

I: Why I put this question to you is that, research has shown that Africa is leading in sexually abusing children. Sometimes people could be using children sexually in secret or others shy away from speaking out or telling their parents?

P: But I have not experienced that in my village

I: So, in this village of Rwamurari what measures have you put forward to succeed in preventing children from being used sexually/defiled, as you have stated it that you are a leader, what have you done? Or else it was done by religious leaders or the army to see that they protect the children?

P: When this habit of child sacrifice rose up and murdering of people, we advised parents to always be with their children or else when a parent wants to leave her child at home, she leaves her with someone older to take care of that child at home. That issue we emphasized it very much in this village such that a child is not left alone

I: As a parent and a leader, what measures have you put up so that when these children are either going or coming from school or going to fetch water on the spring at night or early in the morning are not defiled?

P: We eliminated the habit of children walking at night while going to school children walk with their older brothers or sisters or parents but for a child to walk alone, we try to fight it

I: What about religious leaders, is there a way they are trying to discourage that behavior from happening?

P: Very much

I: What do they say about it?

P: Not to leave a child alone, a child should not move out late evening, all that they talk about it

I: Do they tell people on what to do in case it has happened; I mean like taking a child to the hospital for testing to verify whether indeed the child has been raped or make a police report?

P: All that has been taught to people in my village and if at all it has happened, we rush to the hospital for HIV testing to confirm whether she has contracted the virus or not

I: Now, let’s turn to parents. As a parent what measures have you implored to ensure that your child is not sexually abused/defiled?

P: It is every parent’s responsibility to teach her child mannerisms. Teach her how to behave and to always report to me what troubles her and I also help her.

I: As a parent how do you now that your child is feeling well or bad?

P: She will be happy

I: And how do you come to know that she has been defiled or have had bad touches? How do you know it

P: She won’t be happy and in case she has been defiled, she will be crying or walking abnormally

I: What else?

P: Because when a child is happy, she must be playing

I: Have you taught your children on how to protect themselves?

P: As a parent, my children are always with their mother. Secondly, when a child is not happy I realize it myself and can tell something wrong has happened. I start asking of what has happened to her or how do you feel?

I: Tell me if you have tried to teach your child about sexuality

P: No, because a child of three years can’t be taught about such things

I: You don’t have children aged between nine and fourteen years?

P: No, but my child can’t walk at night or come home late. The other thing I cannot allow my child to dress improperly and if I have protected her that way, then I believe am on the right track.

I: Thank you very much, does your wife usually sit with her girl children to advise them not to engage themselves in sexuality while still young?

P: Yes she always have time for them because things have changed due to COVID 19 even if she is going to the garden, she has to go with them most of the time.

I: Our last question in all that we have discussed or what could you have forgotten and would like to add on because you could have noticed something about yourself.

P: The other thing that should be introduced is caning because when a child is not beaten, he becomes unruly

I: What are your closing remarks?

P: I’ll end by saying that we should cooperate as parents and fight such acts so that these habits don’t multiply amongst our children.

Thank you very much

**Translation 13**

I: What do you understand about sexual harassment?

R: I think its playing sex with the youth without their will

I: How did you know that?

R: I came to know about them from the radio and also being sensitized. You can hear that a father has raped his daughter or you hear that a girl was raped

I: Have such cases ever happened in you village?

R: Yes… in the past days a boy aged sixteen years just made a fourteen year old girl pregnant and now they have a child while they are still young. So you see that is a lot of misery.

I: How about where you find it was a man who impregnates a young girl like that? Have such cases taken place in your village?

R: They are also there. You find that a man is thirty years old impregnates a girl who is like sixteen year.

I: Like how many people do you think have done that in your village?

R: They are like three.

I: Did they begin happening long time or it is when they just began to happen?

R: They were rare. Long time you would find that a man has married a girl who is not yet eighteen years old but they stay together in their marriage.

I: Are there cases where the youth are raped and the parents ignore it and you fail to know about it?

R: They can e there but when we don’t have an idea about them

I: Is there any man who married a very young girl?

R: I have not heard of it. It may be there but when I have no idea about it

I: Have you heard of anyone whom it has happened to?

R: I heard of it outside of our village. I heard that a woman who was forty eight married a sixteen year old boy.

I: What did the leaders do when they heard of the youth who were sexually harassed?

R: I talked to her and I told her to go and report him to the police but it’s like she did not go there. The girl told me that she went and told the chairman LC1 but he did not help her.

I: Why did the girl come to you?

R: I work as the para-social worker for the youth. I saw that the girl was not yet of age so I advised her to go to report to the police. But the mother of the girl and of the boy are friends, so I think they decided to settle the issue amongst themselves and the girl also stopped coming to me.

I: Do you think that in such acts the youth are likely to get infected with HIV?

R: You are likely to get it because you can have played sex with someone that you don’t know their HIV status. In the past days there was a man staying in this center and he was taking HIV tablets and he raped a young girl but the good thing is that they arrested her and right now he is in the hospital, Kyamugorani

I: What measures have you put to make sure that you protect such youth from being sexually harassed?

R: We try to sensitize them. In case I find them in a group I try to advise them to always go home early and to avoid bad peer groups and being lied to. Io advise them so that they can avoid being sexually harassed and we also try to sensitize parents so that they can avoid over sending their children

I: Do you usually sensitize parents or you talk to them once in a while?

R: We usually sensitize parents when we meet each other in SACCOs. We don’t usually meet them.

I: What would you want to be done in order to stop such acts in your community?

R: The people should be sensitized in the village because it is hard to reach them. people that work on such cases should put in more efforts to help those that report such cases

I: Is there anything that you would like to ask connected to what we have discussed about?

R: I request you to advise me on what I can do according to what I have told you so that I can also learn on how I can advise people?

I: *(saying a proverb…)* you should keep sensitizing parents to guide their children to avoid being sexually harassed. They can keep going with them to do different works. You as leaders, you should keep reminding parents that the children need their advice and counselling.

**END**

**Translation 14**

I: Have you heard about sexual harassment among the youth?

R: I usually hear about it. Sometimes I hear that a father has raped the daughter. This thing usually hurts me.

I: What do you understand by sexual harassment of the youth?

R: I think that it means old people or even parents raping their children and this spoils their future.

I: Has it ever happened in your village?

R: It happened in our village, actually my neighbor. They said that a house boy raped a child and they arrested him and put him in prison, he is there up to now

I: How did they know about it?

R: They told us that they house boy was from working he went in the house and raped the child and she screened. When the people heard her screening they came and found the boy dressing up, so they arrested him

I: What did they do after coughing him?

R: They first beat him up but to me I think that they made a mistake of beating him.

I: Do you thing that sexual violence among the youth is likely to make them get infected with HIV?

R: They are likely to get infected with HIV because they don’t know the HIV status of the person who has played sex with them

I: Do you think that there is sexual violence in your village?

R: People fear it because they know that once you are caught, you are arrested and taken to prison

I: As a parent, how do you understand sexual violence among the youth?

R: I know that it is something very bad. I don’t support it at all.

I: What has been done to prevent the youth from being sexually harassed?

R: There are usually monthly seminars where the youth and parents are sensitized about sexual violence. Even VHT usual advice parents and the youth on what to do

I: Do church leaders usually talk about it?

R: They talk about it very much in churches

I: How have you been able to protect your children from ending up in activities of sexual violence?

R: I tell them that we are in difficult times so they should avoid people who give them money. I also tell them that today there are very many diseases so they should try to avoid them and most times I am always with my children and we work together

I: How can you know that your child has been sexually harassed?

R: I can ask her and she tells me

I: What are some of the things that can make parents fail to know what has happened to their children?

R: It is caused by parents failing to befriend your children, so if you even stay away from them, you find that they cannot be free to tell you

I: Do you think that it is nice to tell your children about these issues about sexual violence when they are still young so that they can know about it and be able to avoid it?

R: It is very nice because you can have informed him

I: Thank you very much, could there be any thing that you would like to ask me related to sexual violence?

R: I have no question

I: Okay thank you

**END**

**Translation 15**

I: Have you heard about sexual harassment among the youth in this village?

R: I hear about them from other neighboring villages and also on radios

I: What does sexual harassment among the youth mean?

R: It is destroying their peace and life

I: What do the community members did after a youth had been raped?

R: I think they arrested whoever raped that youth

I: Do you think that in your village there are some youth who are raped but keep quite about it?

R: They could be there. A youth is likely to hide it from you

I: What have the leaders of your community done to help the youth who are sexually harassed?

R: They take those who rape the youth in prison

I: How do you protect your children so that they are not sexually harassed?

R: I protect them. I try to make sure that my children don’t go outside the house

I: Why do you think that there are no sensitizations about sexual violence among the youth?

R: Sometimes the youth fail to say that they have been harassed. But once we find out, we go to the authorities

I: Is there anything that you would like to ask me related to sexual harassment among the youth?

R: No… the most important thing is to try and protect after our children so that they are not raped. We can tell them to be careful and once we have told them, they can do what we have told them to do.

I: Do you think that it is nice talking to your children about sexual harassment when they are still young?

R: Sometimes a child can tell the parent if anything confuses them.

I: What do you do to make sure that your child tells you everything that happens to him?

R: It is according to the things that you do for the child. For example there was a child who was staying with the father but she was suffering since the mother had also gone, so I decided to go and pick that child and look after her.

R: Okay… thank you very much madam for very important thing that we have been able to discuss about and it is going to help us come up with different ways of helping the youth to avoid sexual harassment

**END**

**Translation 16**

I: What do you understand by sexual harassment?

R: There is playing sex. Me I think that that is for old people betwwen the husband and the wife.

I: How did you know that the youth are not supposed to engage themselves in sexual activities?

R: I grew up knowing about it that the youth are not supposed to play sex.our parents used to advise us.

I: If a youth plays sex, what happens?

R: A youth can get very many problems?

I: Do you think that there are some youth who play sex willingly or even not willingly?

R: They can be there

I: How many do you think they are?

R: They may be either five or six

I: Do you think that they can be willing or they cannot be willing?

R: Some of them can have been raped?

I: How many do you think can have raped?

R: I have personally seen two

I: Did they identify those who raped them?

R: They arrested

I: Who caught them?

R: Their parents

I: How did they know that their children were raped?

R: One of them was caught in the action and another one it was the child who reported him to the parent.

I: What did those parents do?

R: They used the police to arrest those who had raped their children

I: What did you as community members do?

R: We helped the parent to arrest those rapist.

I: If a youth is raped, do you think they may get infected with HIV?

R: They can get infected because if the youth plays sex with someone who is HIV positive then they can infect them

I: Did they medically examine the rapist of those youth?

R: They caught them and took them to the police station. They examined them but for us we did not know the medical report.

I: How about those youth, did they take them to the hospital for a medical examination?

R: Yes…their parent took them to the hospital for an examination

I: How long did they take to take those children to the hospital for a medical examination?

R: They took them that day.

I: What measures have you put to make sure that the youth don’t face such problem?

R: To prevent your children from going to far places, keeping them close to yourself and tell them to avoid moving at night.

I: But where did they rape these children from?

R: One was found collecting firewood and another one was raped by the house boy at home.

I: What do the leader in your community leaders talk about sexual violence?

R: The leaders also condemn them a lot. I am also on the LC1 committee, we try to condemn them.

I: How about church leaders?

R: It is also the same with them. They condemn them a lot.

I: You as leader, do you usually teach the youth on how they can avoid such things?

R: As leaders we usually call parents with their children and we talk to them

I: How many times do you do that in a year?

R: Like three times in a year. At the beginning of the year, in the middle and at the end of the year.

I: How did you manage to do it during the lockdown period?

R: We tried to protect our children from being raped

I: What would you want to be done in your village that can help to protect your children?

R: I would want that the authorities arrest that rapist and it becomes as an example to others which will make them fear.

I: Are there some youth in your village who are raped and they keep quiet about it?

R: They are there because you find that a sixteen year old girl is pregnant. So that can be showing that they raped her and she kept quiet about it

I: How many do you think keep quiet about it?

R: In our village, we have around seven girls who became pregnant.

I: In your village do you think that there are parents who don’t care when their children have been raped?

R: They are there. For example there was a parent who hide the daughter after she had been raped and we later saw her pregnant so we knew that she had been raped

I: Eh…

R: They are there and some hide their daughter until you find that they are actually pregnant, that is when you can know that she was raped

I: Thank you very much

**END**

**Translation 17**

**M**: When you hear about sexual harassment, what do you understand?

**R**: Sexual harassment?

M: Umm…

R: The youth have a very big problem and you should help them a lot

M: As a man, what do you understand by sexual harassment among the youth?

R: I don’t understand that issue.

M: Have you never heard about it?

R: I have never heard of it and I have not even heard people talking about it

M: How about when we talk about raping of the youth?

R: I know about that because it has even happened to my own children

M: Your children?

R: Yes they raped them

M: How did you know about it, what happened exactly?

R: My children first spent like two or three when I did not know that they had already been raped and that they had spoilt their private parts

M: How old were they?

R: One was eight years while another was six years old

M: Did they rape them at the same time?

R: Eh… they raped them on the same day

M: So how did you come to know about it?

R: When we saw the children coming while they were not walking properly, we rushed them to the health workers. Then we went to report and the girls said the name of the person who had raped them, we found out that it was one the men that stay in this village so they arrested him and he is in prison

M: He is still in prison?

R: Yes… he is in prison

M: How long has he spent in prison?

R: I think he is going to reach three years while in prison

M: Do they usually rape girls in your village?

R: It’s rare but it was only that man who raped my children but they arrested him

M: How were you able to notice the man who raped your children?

R: We saw that the children had been raped; we asked them who had done it to them and they told us the man’s name. So when we asked him whether he was the one who did it, he accepted that he was the one

M: So who caught him?

R: It was the chairman who arrested him and put him in prison

M: Then what followed?

R: The name was put in prison

M: What did you do to your children?

R: I brought them here at the hospital for a medical examination because the other man was HIV positive. They tested them twice and found that they were not infected and were safe

M: Do you think that sexual harassment is one of the ways in which the youth may get infected with HIV?

R: No… I would not want that to happen but even when they rape them, it’s not good

M: What I mean is that, can a youth get infected with HIV after being sexually harassed?

R: No… that cannot happen after one has been sexually harassed

M: After finding out that the man was infected with HIV, what did they do?

R: They made him to begin taking HIV medicine and that was like the punishment

M: Do you think that such cases are common in your community?

R: They were common but recently the people condemned it and the cases reduced since they arrested those that did it

M: What was done to make sure that such cases reduce?

R: People began fearing

M: What did they fear?

R: They feared that they could be arrested or that even the people could kill them. if they are to do them, they do it at night

**M**: Are there cases where you find that the father of the girl who has been raped just keeps quite about it?

**R**: It can also happen

**M**: As a parent what measures have you put to make sure that this does not happen to your children?

**R**: You just be so protective and make sure that your children stop loitering, you make sure that they stay by your side and as a parent you make sure that you monitor all their movements and you put in efforts

**M**: How do you put in efforts?

**R**: So that they also fear

**M**: What have your leaders done to make such that they protect the youth?

**R**: they began by putting in a lot of efforts but I think they later ignored it?

**M**: How about on the radios

**R**: I don’t have a radio, so there is nothing that I know

**M**: How about at the church, don’t you pray on Sunday?

**R**: Not every day because there is when you go to pray and fail to hear anything. so I don’t know anything

**M**: Thank you very much, unless you have anything that you want to ask

R: No…

M: Thank you very much.

**END**

**Translation 18**

M: What do you understand by the term sexual harassment among the youth?

R: I personally understand sexual violence as a very bad thing. I always advised my children as while growing up to protect themselves about such things. I used to tell them that it is the worst thing ever. To protect oneself against sexual violence, I used to tell them that a child is not supposed to engage herself in sexual activities before the right age of marriage and indeed my children grew up well behaved, I used to tell them that I don’t like them moving around in peoples homesteads anyhow, not to move around in the night, not to sleep outside in other people’s homesteads basically that is how I raised them. My children only become stubborn when they had already matured and moved to far off places but otherwise my children were really raised properly though people think that there is one of my children who got spoilt when she was me.

M: What about the youth that you stay with?

R: I also have them. But I have not had a child who has really messed up in life and wonders off to places where I haven’t sent them to.

M: Where did you learn all this information from?

R:: I was also taught by my parents, while growing up they always reminded me not to roam around just like that, not to sleep in other people’s homes anyhow, Surely I was brought up very well until the time of my give away I did not encounter any problems,

M: Are there any children who have encountered sexual violence in your village?

R: Yes… I have noticed many since three to nine years back.

M: So how many children do you think have been affected?

R: Sure there are so many… sometimes they are on their own and they accidentally come across them, you try to advise them but some of them are big headed and they do not want to listen, sometimes you find you are not together even when you are advising her. But most of them are involved in such activities.

M: How do you understand that a child is involved in sexual activities?

R: In most case you see yourself how these children are being fooled around most boys, sometimes you find they are just playing together in a grazing are alone or when they have gone to collect say fore wood and you really find when they are on top of each other. Don’t you think at that point they have starting engaging in sexual activities? That is how they start slowly by slowly.

M: That is how they start?

R: Umm

M: Umm

R: Just imagine a girl of nine year and boys are all on top of her, is that that how they start getting spoilt.

M: So are there adults in your community who are involved in assaulting your girls sexually?

R: No… we don’t have such people.

M: So have you ever heard of a case where a youth has been sexually harassed?

R: Eh… I usually hear about them on the radio but I have not heard of any case in our community

M: They are not there?

R: Eh… because we have very a good chairman who really fights against such cases

M: Have you ever heard of a youth being sexually harassed?

R: Yes… I have heard of it but I have never seen any case in this village

M: You just hear about it from the radio?

R: Eh… I have heard it on the radios like in Mwizi but they are rare in this community

M: So about what you heard in Mwizi, were the people able to identify the rapist?

R: Eh… they arrested him

M: They arrested him?

R: Eh… even up to now he is in prison

M: How did they know that the girl had been sexually harassed?

R: They got him in the action and when they tested the girl, they found that she was pregnant

M: Among the community members, whom do you think caught him?

R: I am not sure of the person because we also heard about it in the rumors but we know the rapist

M: Do you think that such cases of sexual harassment can make the youth end up being infected with HIV?

R: Eh… that is possible because actually that man who raped her was infected with HIV

M: Eh…?

R: I was not around but that is what we heard that the man was infected with HIV

M: So when they found out that he was infected with HIV, what did they do about it?

R: I don’t really know and I don’t know where the girl is because I stay far away from that place but I know the rapist because he once stayed in our areas, all I know is that he was arrested

M: Did they medically examine the girl?

R: I don’t really know and I don’t want to lie to you because the information just came to our place and I have never seen the girl, so I can’t lie to you that I know where she is

M: You don’t know?

R: Umm…

M: So can you know whether the girl was found infected with HIV or not?

R: No… I don’t know about that but probably the people from her community are aware because they know her

M: You have told me that there is no case of sexual harassment in this village?

R: Umm….

M: Why do you think that these cases have not yet taken place in this community?

R: I think it is the protection that is there

M: How do they protect?

R: Our chairman is too protective and also the people themselves because they know that once they rape someone, they cannot survive imprisonment. So we have never had such behaviors in this village because I have never heard that someone has raped another person’s child

M: Do they sensitize the people or?

R: Eh… they fight against those who smoke marijuana and even those who still do it, they do it in hiding. This helps to avoid cases of sexual harassment because these activities are the common causes of rape

M: As parents who look after young children, apart from advising them is there anything else that you have done to prevent them from getting involved in such activities or meeting these rapist?

R: What can I really do? The only thing that I can do is to protect them and prevent them from visiting and loitering at night. I teach them good morals so that they can inform me of where they are going before they leave; I sensitize them about the kinds of people that they should avoid visiting so that is how I protect them

M: How about the church leaders and the different leaders in your community, what do they have to say about sexual harassment of the youth?

R: They also advise them though not very much, but they sensitize parents during mass to protect their children so as to prevent them from being sexually harassed or even being stolen from them

M: Umm…

R: They also teach us to regulate the movements of our children so that they don’t fall into danger

M: Is there anything that you would wish to be done in your community that is in line of sexual harassment among the youth in your community?

R: Now what can I say because everyone has their own different ways, so I would call upon the leaders to continue sensitizing us on how to protect the lives of our children because personally I could be protecting myself but there are some people who don’t know what to do.

M: Umm…

R: So we need counselors who can also come and sensitize us in our different centers because there are people who are always drinking alcohol and don’t know how to protect their children

M: Umm…

R: So we need people to sensitize us so that we get informed and learn how to also sensitize our children, so we need such services but we don’t have them

M: In the past months, children have not been going to school, how have you protected them?

R: We protect them by staying with them at home and if it means going to dig with them, then go with them. Just make sure that the children are busy all the time but if you spend all your time in clubs drinking alcohol then you are not protecting them

M: So could there be any question that you would like to ask me connected to sexual harassment among the youth?

R: Eh… What other advice can you give us to add on the above?

***… (Audio ends abruptly toward the end of the conversation.)***

R: In most case our youth do not mind at all about their lives… it is like when they engaged in such issues, it is as if they are there is something behind that is motivating him or her to do that. So if we can get such sensitization meetings, I believe there is a way that they can start looking at this world in a positive way and behave… they also understands that what they are doing is actually wrong.

M: You mean there are also parents who do not mind about such children are doing?

R: Umm… there are there and they do not care… even if they are told that his or her child was caught somewhere having sex, you will find that they are very far away from that. They do not even sad about what happens to them and sometimes you find it the parents themselves who are actually supporting them. Such things are so painful but if you come and sensitized, you will get a few who will pick up something and the parents will be in a position to advise their children just in case.

M: But the issues of sexual violence in your community are not so common?

R: For sure they are not common… unless those go there willingly with his or her conscious and when he or she is interested. But if girls also mess around with boys, definitely they will be raped. Sometimes these boys also are not a joke because in most case they are not in their right mind after using drugs. If they come across them, they can easily rape them.

M: Okay… we are ending here our discussion.

END

**Translation 19**

M: Like I had already explained to you about sexual harassment among the youth.

Have you ever heard about sexual harassment among the youth?

R: To hear about it?

M: Umm… Have you ever hear of it

R: I have actually not heard of it but I have real seen it at my own home

M: At your own home?

R: Umm… at my own home

M: What happened?

R: They raped my daughter

M: How many youth in your village have been sexually harassed?

R: I heard of them and they were around five

M: Until it also happened to you as a parent ?

R: Umm…

M: Are these cases still taking place or they reduced?

R: It reduced and I have not heard of it again

M: What do you think causes sexual harassment among the youth?

R: I think that it is caused by the people who smoke marijuana because when you look at the young child then you know that whoever did this was not mentally stable

M: Apart from being raped, do you think that there are other ways of sexual harassment among the youth?

R: Umm…

M: Like which ones?

R: I think if a parent fails to properly look after their child and they begin to desire different things which may make them engage in activities that they are not supposed to do

M: Is there anyother form that you know?

R: No…

M: What do you think made your daughter to be sexually harassed?

R: I also did not understand it because she had gone up on the hill to fetch some fire wood and she came back home while bleeding. So I don’t really know what happened

M: Where you able to identify who sexually harassed your daughter?

R: Yes… I identified him and he was even arrested

M: Who arrested him?

R: He was arrested by the chairman LC1

M: At that exact time or it was after some days?

R: He raped her in the evening and we arrested him in the morning

M: Where you able to get any help from the community members or even the leaders in your village?

R: They helped me because they arrested the man and took him to prison, then took the girl to the hospital for medical examination

M: Were they able to know the HIV status of that rapist?

R: Umm…

M: Did they medically examine him?

R: Umm… they examined him

M: So how did they find his HIV status?

R: They found out that he was not HIV positive

M: How about the girl, did they examine her?

R: Umm… they examined her and found out that she was also not HIV positive

M: Do you think that sexual harassment among the youth may make them get infected with HIV?

R: Yes… I know that it can make them get infected with HIV

M: How?

R: I know that a youth may be sexually harassed by an infected person and sometimes you may not even know that your child has been sexually harassed so that you can take her for a medical examination

M: As parents what things have you done to make sure that your children are not sexually harassed?

R: You make sure that you protect your child all the time

M: How do you protect her?

R: To make sure that she is by your side and you do everything you can to give her what she wants

M: What do church learders and government workers say about sexual harassment among the youth?

R: Most times they sensitize us and condemn it

M: How do they condemn it

R: They say that sexual harassment destroys young children

M: As parents, don’t you sensitize your children about sexual harassment?

R: We sensitize them to avoid being around some people so as to protect themselves from being sexually harassed

M: From the time your own daughter was raped, is there any other youth who has been sexually harassed that you have heard of?

R: No… there is no one else who has been sexually harassed because the rapist who sexually harassed my daughter was a worker from Rwanda, when they arrested him I think the other workers also feared

M: Is there anything that was done to prevent men from sexually harassing young girls in your community or nothing was done?

R: There was nothing done because all they did was to arrest the man, I have never of the chairman gathering people to seisitize them about sexual harassment

M: What do you think made this man sexually harass your daughter?

R: I did not understand the reason and up to now I have never understood it, I even keep thinking about it in my head but I have failed

M: What measures have the leaders like the LC1 and the chairman among other put across to reduce on the rate of sexual harassment among the youth?

R: I have not yet seen anything

M: You have not seen any?

R: Umm…

M: Even on radios there is nothing they talk about protecting the youth

R: I have on radios different people sensitizing the youth on what to do but in our village, I have not seen the chairman or even the counselor sensitizing anybody

M: Eh…

R: Umm…

M: From the time that incident took place in your family, what have you done as a mother to make sure that you protect your other young children?

R: I tried to avoid sending them to difficult places, now once it is late I cannot send my child to go and fetch water but instead I go and fetch it myself. Because my daughter had goen to collect fire wood and they raped her from there since it was even already 7:00pm

M: 7:00pm?

R: Umm…So from then I tried to avoid it so now even if there is no water and its late, I go and fetch the water myself

M: Umm…

R: So it is now rare to ind me sending my child to difficult places, I go there myself and in case I send her and it becomes late when she has not yet return, I follow her

M: Is there any suggestion that you may think should be enforced in your community that is connected to sexual harassment?

R: I first thought that maybe our community has bad leaders because if you are to see what the youth are now doing, most of them have failed to study and the parents themselves are always drinking alcohol and fail to care about their children.

M: Umm…

R: So I think that parents should be sensitized to care for about their children and take them to school because you find that a parent goes to the bar in the morning and they don’t know whether the children have gotten what to eat or not

M: Umm…

R: Eh…

M: Thank you very much.

**END**

**Translation 20**

M: What do you understand by the term engaging children in sexual activates?

R: I think that is when you push your child to get married before the right time.

M: Umm

R: Allowing a child to get married before the right time to get married.

M: Where did you first hear such a thing?

R: I always year people speak about such information on the radio talk shows and programs

M: Do you really think such think happen in your community?

R: They have never happened in our community.

M: You have not heard anyone speak about anything that such things do exist?

R: Not at all…

M: What about men raping and defiling young girls?

R: *(Silence… )*

M: It has also never happened?

R: Umm

M: But you mentioned that you have ever heard of such issues of sexual violence on the radios?

R: Umm

M: What did you hear exactly, did they arrest the perpetuators or not?

R: But for sure, personally I do not usually follow such things

M: Why do you think that such issues of sexual violence have never reached your community?

R: They just meet innocent young girls on the way and they rape them… and that is all. Young just hear people say that a child has been raped… just imagine an old man of 30 years by forcefully having sex with a child of say 7 years. But such actions have not yet been seen to happen in our community.

M: They have not happened?

R: Not at all.

M: But why do you think such think are not happening in your community?

R: They are not there as of now but may be they will be happening with time.

M: You think they will be happening with time.

R: Umm.

M: Are they some things your community leaders are doing to prevent such men not to engage your children in issues of sex?

R: Not at all, may some of those girls who work in bars and you can really see that they are not yet that age of working there and they are already engaging in sexual activities with very old men.

M: Those girls are more than 14 years of age.

R: In fact some of them are beyond 14 years of age.

M: Our focus is on very young children

R: Sincerely such things behave not happened in our community especially in these days.

M: It may not have happened in your community but you as parents are there some measures that you have put forward to prevent your children from engaging in such activities?

R: May be you just advise them to avoid engaging in such risk behaviors and we tell them the dangers involved.

M: Only that?

R: Umm.

M: So now that it has not yet happened, but we thank God… but to avoid such thing from happening in future what would be your wish to have such issues to be sorted?

R: You cannot avoid certain things if they are to come because they are men out there who use drugs and alcohol …. And you never know in future they can end up doing such things.

M: That is the reason I am asking you?

R: Umm

M: There is a saying that “prevention is better than cure?

R: Umm

M: What do you think can be done to prevent such things from happening in our communities or to prevent our children from engaging in sexual activities?

R: I think we need to be sensitized so that people are aware about is going on and how they can overcome such challenges.

M: But such things have never happened in your community?

***….. Recording stopped …then another recording resumes***

M: You told me that some of these things have never happened in your community**?**

R: Umm.

M: How come such things are not happening in your community?

R: I think they are so protective…

M: They are protective?

R: Umm

M: Protective in which way?

R: They fear

M: Who fears… the children or adults?

R: Even the adults.

M: Eh…

R: I have never heard of a man defiling a child in our community.

M: Do you think such actions can easily lead such children to get infected with HIV.

R: Umm

M: In what way?

R: You get such boys who used drug substances when they rape our young girls, you may find they are already infected and he ends up infecting this innocent child.

M: But as for you, you do not know why such things have not happened in your community?

R: Nothing at all.

M: Even your community leaders have not said anything at all about it?

R: They would have said anything if there was any case that has happened already.

M: So because nothing has happened, they do not have anything to say about them.

R: Umm.

I: Okay thank you very much.

**END**

**Translation 21**

I: Like I had explained to you what it means by sexual harassment, have you ever heard of it and what do you understand by it?

P: Sexually harassing young girls?

I: Umm…

P: I think some men gives young girls money so that they play sex with them, so I this sexual harassment is brought about by the too much love for money among the youth

I: Umm….

P: You find that their parents fail to tell them that it is wrong getting money from men and so they grow up thinking that money is more important than their own lives

I: So giving the youth money is one of the tricks of sexually harassment?

P: Eh…a girl is lied to and they end up being sexually harassed without having a crew about it, so when a man has failed to get what to use to convince the girl, they resort giving them money and they also accept .

I: Have you ever heard of a case of sexual harassment in your village?

P: I have never heard of it in this village but I have heard of it else where.

I: From the places where you heard of it, where they able to identify the girl and the rapist?

P: Umm… they identified them and actually arrested the rapist though I am not sure whether he is still in prison but I am sure that he was arrested. Who they came to know about it they found the girl pregnant and asked her who had impregnanted her so she mentioned the name of the old man whom they arrested.

I: What did the village people and the authorities do after hearing about the case or it was left as a responsibility of the father and mother of the girl?

P: By that time the girl was staying with only the mother because the father was working from kampala but she put in a lot of efforts and went to report to the police.

I: Umm…

P: The authorities just helped her to arrest the rapist

I: Do you think that sexually harassing girls makes them get infected with HIV?

P: Very much

I: Very much?

P: Umm…

I: How?

P: If a woman who is HIV positive rapes a young boy she has the intention of infecting her with HIV and that is the reason to as why she cannot even try to use a condom.

I: Umm…

P: Even if it’s an old man, the young girl cannot request him to bring a condom because she is afraid of him.

I: Umm…

P: So because he has more energy, he ends up raping her and in that process she cannot fail to acquire HIV

I: Has such a case ever happened and you find that a girl after being sexually harassed she has acquired HIV?

P: I have never heard of such a case

I: You have never heard of it?

P: But in my own understanding, I think that if a man is HIV positive and he rapes a young girl, she much also get infected

I: Umm…

P: Uh…

I: You have told me that you have never heard of any case of sexual harassment in your village?

P: Umm…

I: Do you this that there are cases where the youth are sexually harassed?

P: They may be there because I don’t really know everything that takes place but such things also come up

I: How can you personally know that a girl has been sexually harassed?

P: The youth of these days are not easy because a girl can be raped and they stay silent about it and you can never suspect that they were even sexually harassed

I: Umm…

P: Its not until you begin to notice some chages on the way she conducts herself or probably you send her to buy something and she delays, so that’s when you ask her and try to find out what is wrong

I: Umm…

P: But the youth of one days cannot tell you

I: They cant tell you?

P: Umm…

I: As parents what measures have you up a place to make sure that your children are not sexually harassed, you find that you spend the whole day riding the motocycle while your child is home alone. So what measures hve you put across?

P: You tell the mother to make sure that they protect the child because in most cases it is the woman who looks after the children since we men are always busy working and never at home

I: Umm…

P: Because it is difficult for one to rape her when its still daylight because everyone is seeing and by the time its 7:00 pm you make sure that you have already command your children to be home and avoid cases of walking at night

I: Umm…

P: So I think that it would be an easy job to care for your children and monitor their movements and be strict on them to avoid walking at night

I: You monitor them?

P: Umm…

I: What have leaders forexample church leaders and even whose in offices done to sensitize the people about sexual harassment?

P: I think that the only thing that the churh leaders can do, is to encourage the parents to bring their children to the church every Sunday so that they are taught good morals

I: Umm…

P: But I have not heard of such cases of the youth being sexually harassed because we have churches were parents can be taught of what to do to guide their children, they tell them that they should not come to pray and leave their children at home because they can learn a lot while there

I: Okay… apart from you the parents teaching your children, is there anything that the authorities have done to sensitize the youth about sexual harassment?

P: No…

I: Eh…

P: Umm…

I: Thank you very much. Maybe if you have any other question..

P: there are cases where you find that your child has been sexually harassed but the rapist has more money than you, so once they are arrested they pay bribes to the authorities and he is released from jail quickly

So what offices can we contact to help us with such cases so that as a low income earner you are helped

I: Umm…

P: Uh…

END

**Translation 22**

M: So now you as a person, how what do you understand by sexual violence, how can you explain it?

R: I know that it is a very bad thing

M: No but if I say that engaging children in sexual activities, what do understand, how can you explain it or what is it?

R: It means involving me in issues of sexual activities and finally having sex.

M: I request you to speak a bit loud

R: Umm.

M: So where did you first hear of such things?

R: I heard it from my own parents

M: From your parents?

R: Umm

M: So in your area *(Rwibogo…)* do you think such things are common there.

R: You mean a situation where by people in our community to engage children in sexual activities?

M: Umm

R: In most case such people are porters especially when they are on their own grazing and they are alone. Or they have gone to fetch water at a well… that is how they end up doing such things

M: That is how they get involved while they are still children?

R: Umm

M: So you are saying such thing happen in most cases?

R: They happen but it depends on the time and the movements of such children they make or when they move, they have any person who is mature and older than them to protect them.

M: Have you ever heard of a child who has been sexually assaulted in your community?

R: Yeah…

M: Who first heard of such news?

R: A person who will know about it of course is a parents because they are some people after raping … they really injure their private parts badly and sometimes the child keeps quiet. But because a parent has an eager eye, she or he looks at the child and know that something is not right. She may start having difficulty is walking and if you ask her to find out what is wrong with her. That is when you discover that she has been raped.

M: What do they normally do to the perpetuators of rape?

R: Some of them are arrested, put in prison and if you fail to identify them just because the child has failed to disclose his identity.

M: Do you think some of those victims of are rape are at a high chases of getting infected with HIV?

R: Infecting them with HIV?

M: Yeah

R: Yes it happens… even my own daughter you are seeing here, I tell her the truth that ,most of those men who come to telling issues to do with sex, they are all infected. If you accept and have sex with them and they add their HIV virus on the one you have, you will die soon. Yes I tell her.

I: What do they do to men who are involved in such issues of sexual assault…. do they ever get tested about their HIV status?

I: There are some who mind about HIV status but depending on the parent and other they just do not mind and they instead ignore.

M: What about children do they also get tested or they just do not mind also?

R: If you complained that the child could be suffering probably they would get motivated to test her. But it is not usually done.

M: So what have you tried to do, you as parents or the community leaders to event your children from getting sexually harassed?

R: It is only you who take care and the trouble and to continue telling your child about how to protect herself from such things. So you tell her the disadvantages and the dangers of getting involved in such things. If the child is obedient, she will pick one or two things and she will fear such and if she is not, she will go ahead and do whatever she wants.

M: But it has never happened to your child?

R: This one here?

M: Umm

R: No it has never happened to her.

M: Umm

R: Umm

M: Is there anything that you would like to be done in your community but they is no one to do it, Or what you would want your community leaders/ Church leaders to do not instead they are doing nothing about it?

R: For the community leaders

M: Umm

R: Now if you fine a person complaining that her or his child has been raped, then another one bribes that family so that they keep quiet and they ignore the case and if you tell them that the child is not getting any better, they will not take you serious. So it is up to you to take your child for treatment, have her treated and them she gets okay. Sometimes you may find she has been infected with HIV when actually she was not. Such things are there and they are common.

M: But from what you see, like how many children in your community that have gone through such challenges?

R: A few of them are the ones who go through such experiences but the rest do not.

M: Like how many?

R: In a cell for example you can get about five or ten children who have gone through such experiences but they also report to the chairman LCI and they sort’s things from there before the entire public gets to know.

M: Thank you very much but before that, is there any question that you would like to ask me.

R: Umm

M: Okay ask me.

R: We hear that in the hospitals there some medicines that prevent people from getting infected with HIV… it is true.

M: Yeah they get such medicine but only if you explain to the doctors the truth, they give such medicines.

END

**Translation 23**

M: Now like I had mentioned what do you understand by the term sexual violence?

R: What I understand by the term sexual violence, it is when a child is engaged in such things sometime she is affected psychologically and even in her future tomorrow gets affected. According to my understanding, I really see that it is not a good thing at all to find a youth already being engaged in such activities.

M: But according to you how do you under stander by the term sexual violence?

R: Generally it when you find a person’s rights is being stepped on, for example forcing someone into something that she is not supposed to engage in.

M: Where did you first hear about it?

R: Personally, I had it from my own child, I had send my child to go and fetch water for me from the water well and that well was somehow very far, so when she delayed to come back, I sent her brother and I told him to go and look for him. So when he reached there, he found her sister yelling and crying all over, after they had finished raping her. Her brother came back carrying her and that is when I saw it with my eyes but I used to just hear it from others.

M: So how many people do you thing go through that same experience of rape in your community?

R: For sure they are there but in most cases such things happen to the most vulnerable population who are not financially stable. So when the perpetuator is arrested, sometimes the parents do not mind following case and sometimes they deceive them around with some money and the matter just disappear from nowhere. So in most cases you find that they have not even gone ahead to report their matter to the police station or even if you decide to go and report there, they will ask you some money which you do not have and you end up confused and in the middle of nowhere.

M: Do you think you are able identify that particular person who raped your child , who actually hunted and identified him?

R: I identified him after personally I looked for him… I first asked my child whether she knew him and she told me because he was one of the porters working my in laws. So we went there and she was able to identify her. Then after we went to the chairman and we had him arrested… finally he was put in prison.

M: Do you think such actions can easily lead to victims getting infected with HIV?

R: For sure.

M: Did you try to find out about the health status of that perpetuator, and did you go ahead to have the child tested?

R: Yes. We also had the child tested.

M: What was the status of the perpetuator and the child as well?

R: The perpetuator was infected with HIV but the child was not.

M: So when you found that the perpetuator was HIV infected, what did you do?

R: He was arrested and eventually transferred to Mbarara prisons. Whatever happened, I would travel to see how it goes but later they eventually later told me not to go there all the time. After a year, I was just there… I went and approach one of the officers who was working there and he also advised me that it no problem for me to continue going there and see the person arrested. Because my in law used to go there alone and he could request me to withdraw the case and the man comes out of the prison by later, I got demotivated. But I had to make sure that cared for my child gave her all the necessary treatment that she deserved but it was not easy for me and I really got disturbed.

M: But such things are still there in our community?

R: There are still there in the community.

M: What did measure did people in your community and community leaders put help victims of such circumstances.

R: Our community leaders these days do not mind any more, if you as a person do not mind and you do not really put in a lot of effort about yourselves, you will never get the justice that you deserve… they will ask you money and you will not be having it.

M: Now, you as a parent what measures have you put in place to make sure that you have provided security so that they do not go through sexual violence again?

R: I do stay with them but if they go to school, I have to make sure that I discuss with the school administration so that she does not go without their knowledge and when she come back home, I also tell her…. *(Recording just ended…)*

**Translation 24**

M: Like I had told you about sexual harassment, what do you understand by sexually harassing a youth?

R: I understand it that there are some youth who get sexually harassed when they are still young and I think that it is not right

M: Umm…

R: As a parent you are supposed teach your children that it is bad to get sexually involved at a young age because once they have been sexually harassed they are likely to acquire certain diseases like HIV and cardida among others

M: Umm…

R: So I would tell them about such diseases and advise them to be careful inorder to avoid being sexually harassed

M: How can you know that a youth has been sexually harassed?

R: Because I stay with children, I understand them very well so when I am going to work I tell their mother to make sure that she protects them because once these children begin forming groups, they may end up doing wrong

M: Umm…

R: Uh…

M: Where did you get this information from or how did you learn about it?

R: When we found out that we were infected with HIV, they decided to teach us on what we can do and teach our children so that they know how to behave and be able to gain a lot in future

M: Have you heard about some youth who have been sexually harassed or heard about men who sexually harass the youth?

R: Eh… they are there because you find that a mother wears very short clothes and even encourages the daughter to do the same and once you try to tell her that what she is doing is not right, she thinks that you are wrong

M: Have you heardof any case where a youth has been sexually harassed in this community?

R: Eh… of course such cases are common in the villages

M: Like how many forexample?

R: Around six or seven and they give the village a bad image

M: It gives it a bad image?

R: Umm…

M: Uh… among those seven cases let us select one

R: Umm…

M: When it happened, were they able to identify the rapist and the youth who had been sexually harassed?

R: Eh… they knew about them and they ran to the police and it would help if you had taught the youth about it before

M: Who first notices and identifies that a youth has been sexually harassed?

R: When the father learns of it and if the mother says with the mother, she runs to the police to report which may not very much help her. So in that she destroys the good image of the village because she should have taught her children about it a long time ago

M: How did they come to identify the rapist?

R: They later came to know him because the girl mentioned his name, so they arrested him and he is in prison

M: Eh…

R: Umm…

M: What do the village members do when such cases take place, do they leave it all in the hands of the parents or?

R: It is at first very difficult to explain to the community members about what has happened because some of them just laugh about it while other work so hard to make such that it does not happen again.

M: Umm…

R: So the people do not cooperate, a village stays a village because some people stay in their own businesses

M: Do you think that sexually harassing the youth makes them get infected with HIV?

R: Yes… because once a youth has began playing sex, they may end up landing on someone who is HIV positive

M: Apart from that, what I mean is that… if a youth is sexually harassed are they likely to get infected with HIV or there are no chances?

R: The chances are very many

M: Among the cases you have told me about, has it ever happened that a youth who was sexually harassed ended up being infected with HIV?

R: There was a man who raped a girl and when he was taken to the hospital they found out that she was HIV positive but they are still examining the girl to see whether she is also infected, they have reached around seven times while examining her so as to find out whether she is safe or not

M: Umm…

R: Eh…

M: Apart from arresting this rapist, what else did they do when they found out that he was HIV positive?

R: At first this rapist tried to bride the father of the girl by giving him one million shillings but one of the people who were around opposed it and rushed to the police to report the case, so they arrest the man and put him in prison

M: The rapist tried to bride the father of the girl?

R: Eh… they had wanted to just solve the issue by themselves but one wise man insisted and called the police with the help of the chairperson, so they arrested the rapist and put him in prison

M: They arrested the rapist?

R: Umm…

M: Do you think that the cases of sexual harassment of the youth are soo rampant in this community?

R: The cases could be very few but when one cases comes up, it destroys the image of the whole village but once you run to the police to report them, such cases reduce

M: The cases reduce?

R: Umm…

M: What has been done in this community to prevent the youth from engaging in such activities alot?

R: In this community if the youth had jobs thn they could be busy

M: I am talking about those youth who aged from nine to fourteen years?

R: Umm…

M: As parents and leaders in this community, what measures have you put across to make sure that you protected your children?

R: The parent makes sure that their child is always by their side so that they avoid loitering in the shops. When you are going to dig you make sure that you go with her, if its fetching fire wood then go with her, unless she is at school

M: Umm…

R: Uh…

M: Like now in this covid season children where not at school, even you parents you were not at home so the children go tired of school. So what measure can you put across to make sure that you protect your children?

R: During this Covid period, you would make sure that your child is busy so that they avoid loitering around where they may end up engaging in activities and end up acquiring different diseases

M: What have church leaders and government workers done to inform people on how these activities are dangerous?

R: The leaders organize seminars where they tell us that everyone should protect their children until when the covid situation is stable and the children go bad to school

M: Where these seminars taking place?

R: Yes… the chairman was organizing them even when gathering of people was not allowed, he would call few people, so one would also go and teach a nearby frined about what they have taught them

M: Personally, how have you taught your children?

R: During the covid season, when loitering of children had become too much, I went and bought a television and told them to atleast stay there watching. When I am from working I come and find them watching and I also told them to avoid loitering around, I encouraged the old boys to look after their young sisters so that they stop loitering around

M: So that they stop loitering around?

R: Umm…

M: Have you taught them on what they can do in case such a thing happens to them?

R: I teach my children avoid becoming infected with HIV like us and I tell them that in case such a thing happens to them, then they should approach me and explain to me how it took place

M: Thank you very much sir, maybe if you have question that you would like to ask me?

R: What can I do for my child in case I find that she has become infected with HIV?

M: Do you mean after being sexually harassed or from other ways?

R: Forexample you find that your child has escaped from you and you hear news that she has been sexually harassed, so I could bring her here for medical examination

*….* ***(The audio ends abrunptly but it was about to end.)***

**Translation 25**

M: I request you to think about the term sexual harressment, when you hear about it, what do you think it mean?

R: The father of my children died, my first born was four years old and the second was seven months so I looked after all my children alone, I cant say that we don’t have relatives but they are always busy so that is how I ended up looking after them alone from nursery and now one of them is in senior one

M: Umm… But have you ever heard about sexual harassment among the youth?

R: No…

M: You have never heard about it?

R: Umm…

M: How about if I talk about the youth engaging in sexual activities while still young, do you understand it?

R: The reason to as why I have never heard of it is because I have stayed with my children and there is nowhere else they go, so without lying to you I have not really seen them heard of anything bad about them

M: Umm…

R: Uh…

M: But have you ever heard anyone talking about someone sexually harassing a youth?

R: Eh… I have heard about such cases of sexual harassment from other communities but I have not heard of anything from this village. We have not yet had any cases of sexually harassing the youth

M: About the story that you heard about where they sexually harassed a youth, were you able to know whether they arrested the rapist or you did not hear about it?

R: I hear about such cases from the radio where they tell you about a youth who was sexually harassed and that the rapits was arrested, but I have not heard of such cases here in this community

M: Do you think that once a youth has been sexually harassed, she is likely to get infected with HIV?

R: I think as a parent if you are HIV positive and use a razor blade to cut your nails, if your child also uses it then they will also get infected

M: Umm…

R: If y and you share a cup with your child then she is also likely to get infected

M: Umm…

R: Uh…

M: So you think that once a man rapes a girl, she cannot get infected with HIV?

R: No… she can get infected because he may harm her because it is not right for an old man to play sex with a young girl

M: Umm… it is not right

R: Uh…

M: Do you think that there are cases of sexual harassment among the youth in this community?

R: No… they are not there

M: They are not there?

R: Umm…

M: What do you think can be done to prevent the youth from being sexually harassed?

R: I could have told you about it if I had seen its victim and see what they had done to guide her

M: Umm…

R: But if one gets involved in such a case then it will affects her

M: So you have told me that you have never heard about sexual harassment?

R: Umm…

M: What measures have you as parents put across to prevent your children from being sexually harassed?

R: As parents, you tell your child to avoid men because once he meets you and rapes you then you become pregnant and infected with HIV

M: Umm…

R: So you tell her that once they meet them, they should distance themselves from them and avoid them

M: Umm….

R: Eh…

M: How about the leaders in this community forexample the church leaders and even those in government, what do they say about sexual harassment?

R: They also say the same thing and sensitize people to avoid sexual harassment because it is bad

M: Do they be telling the youth?

R: No… for the children they encourage them to go to school and study because it will teach them good morals. So the youth also begin from there

M: They start from there?

R: Umm…

M: Eh…

R: Umm…

M: Could there be any question that you would like to ask me which is commented to what we have been discussing about?

R: Now what I wanted to ask…

M: Umm…

R: How can I teach and explain well to my child about this sexual harassment so that they may also understand it?

***… (there recording just stoped abrunptly)***
